# IL-6 promotes PD-L1 expression in monocytes and macrophages by decreasing protein tyrosine phosphatase receptor type O expression in human hepatocellular carcinoma

**DOI:** 10.1136/jitc-2019-000285

**Published:** 2020-06-24

**Authors:** Wenjie Zhang, Yang Liu, Zhongyi Yan, Hui Yang, Wei Sun, Yongliang Yao, Yun Chen, Runqiu Jiang

**Affiliations:** 1 Department of Hepatobiliary Surgery, The Affiliated Drum Tower Hospital of Nanjing University Medical School, Nanjing, China; 2 Department of Immunology, Nanjing Medical University, Nanjing, China; 3 Department of Hematology, The first affiliated Hospital to Nanjing Medical University, Nanjing, China; 4 Medical School of Nanjing University, Nanjing, China; 5 Department of Clinical Laboratory, Kunshan First People's Hospital, Affiliated to Jiangsu University, Kunshan, China; 6 Jiangsu Key Lab of Cancer Biomarkers, Prevention and Treatment, Collaborative Innovation Center for Cancer Personalized Medicine, Nanjing Medical University, Nanjing, China; 7 Research Center for Clinical Oncology, Jiangsu Cancer Hospital, The Affiliated Cancer Hospital of 15 Nanjing Medical University, Nanjing, China; 8 Jiangsu Laboratory of Molecular Medicine, Medical School of Nanjing University, Nanjing, China

**Keywords:** gastroenterology, liver disease, immunology, tumours

## Abstract

**Background:**

We have previously discovered a relationship between the low expression of protein tyrosine phosphatase, receptor type O (PTPRO) in tumor-infiltrating T cells and immunosuppression. The aim of the present study was to investigate the relationship between decreased PTPRO and increased programmed death ligand 1 (PD-L1) in both the peripheral monocytes and tumor-infiltrating macrophages of human hepatocellular carcinoma (HCC).

**Methods:**

The expression and correlation of all the indices were explored in monocytes and tumor-infiltrating macrophages within both human and mice HCC. The mechanic regulations were studied by using both in vitro and in vivo studies.

**Results:**

We found a significant decrease in PTPRO in HCC peripheral monocytes that was associated with increased PD-L1 expression in peripheral monocytes and tumor-associated macrophages (TAMs) in HCC. Monocyte PD-L1 and PTPRO therefore could serve as valuable prognostic indicators for post-surgery patients with HCC and were associated with increased T-cell exhaustion (Tim3+T cells). A depletion of PTPRO promoted PD-L1 secretion in both monocytes and macrophages through the JAK2/STAT1 and JAK2/STAT3/c-MYC pathways. Increased IL-6 expression was associated with activation of JAK2/STAT3/c-MYC and with decreased PTPRO expression through the STAT3/c-MYC/miR-25–3 p axis. Monocytes and TAMs showed significantly increased miR-25–3 p expression, which could target the 3′ untranslated region of PTPRO. The miR-25–3 p expression positively correlated with serum IL-6 levels, but inversely correlated with PTPRO in HCC monocytes. IL-6/STAT3/c-MYC activation enhanced in vitro miR-25–3 p transcription and decreased PTPRO, while further promoting PD-L1 secretion. Adoptive cell transfer of c-MYC/miR-25–3 p–modified monocytes promoted tumor growth by downregulating PTPRO and causing a PD-L1–induced immunosuppression in an orthotopic tumor transplantation model.

**Conclusions:**

Increased serum IL-6 downregulated PTPRO expression in HCC monocytes and macrophages by activating STAT3/c-MYC/miR-25–3 p and by further enhancing PD-L1 expression through JAK2/STAT1 and JAK2/STAT3/c-MYC signaling.

## Background

Programmed cell death ligand 1 (PD-L1; B7-H1) has been identified based on its similarity to other B7 superfamily members.^1^ The B7 family is broadly expressed by immune cells, as well as by T cells, B cells, macrophages, and dendritic cells, and their expression is upregulated after their activation.^2^ PD-L1 is a negative inflammation regulator that causes apoptosis of activated T cells by binding to its receptor PD-1, and depletion of PD-L1 in a mouse model has been shown to cause autoimmune diseases.^3^ The interaction between PD-L1/PD-1 predominates both in vivo and in vitro in the suppression of T-cell function, and especially in a tumor microenvironment (TME).^2^ Accumulating evidence indicates that PD-L1^+^ tumor cells and other cells in a TME can induce apoptosis, energy decrease, and functional exhaustion in T cells.^3^ The T-cell exhaustion associated with PD1/PD-L1 is a characteristic of many types of human malignancies, including non-small cell lung cancer, melanomas, gastric cancer, breast cancer, liver cancer, and leukemia, and novel treatments based on PD-1/PD-L1 have been developed.^4^ The elimination of T cells, and especially effector T cells, by PD-L1^+^ tumor cells can be blocked by anti-human PD-L1 or anti-PD1 monoclonal antibodies.^3^

PD-L1 is also expressed by immunocytes found within the TME, including lymphocytes, macrophages, and dendritic cells.^3^ We obtained preliminary data showing that PD-L1 is expressed by peripheral monocytes, in accordance with other findings for malignancies.^5 6^ Tumor-infiltrating macrophages (TAMs), which originate mainly from peripheral monocytes, also showed high PD-L1 expression. Macrophages are one of the important antigen-presenting cells (APCs) that play a central regulatory role in TME modulation. Recently, a few studies revealed that APCs could modulate TME by secreting PD-L1.^7^ Compared with tumor-derived PD-L1, APC-derived PD-L1 might be more effective: (1) PD-L1 was negative in some malignancies^8^; (2) APC-derived PD-L1 can also induce immune escape by binding to T cell PD-1^7 9^; (3) APC-derived PD-L1 was more effective in preventing T cell infiltrating into tumor^8^; (4) Tumor cell–derived PD-L1 by IFN-γ stimulation was transient, while the APC-derived PD-L1 was stronger, more durable, and partially IFN-γ independent.^10^

The JAK/STAT signaling pathway is a classic pathway that links inflammation to tumorigenesis. Activation of JAK/STAT signaling promotes tumor growth and invasion as well as immunosuppression.^11^ Moreover, PD-L1, one of the hot spots in current immunotherapy, was reported to be regulated by JAK/STAT signaling in many cell types, especially JAK2/STAT1 and JAK2/STAT3.^12^ However, one group reported that PD-L1 was also regulated by interferon-gamma–JAK1/JAK2–STAT1/STAT2/STAT3–IRF1 axis in melanoma cells.^13^

Physiologically, JAK2/STAT3 is commonly transition activated and can be inactivated by dephosphorylation of its upstream kinase by phosphatases, including small heterodimer partners and suppressors of cytokine signaling.^11^ Previously, we reported that protein tyrosine phosphatase, receptor type O (PTPRO) and its truncated form (PTPROt) were negative regulators of JAK2/STAT3 activation.^14^ A relationship has also been established between PD-L1 expression and the activation of JAK/STAT signaling, as activation of JAK2/STAT1 signaling was reported to regulate PD-L1 expression in tumor and immune cells. Therefore, we considered the possibility that a decrease in PTPRO expression might promote PD-L1 expression following IFN-γ stimulation. However, previously reported values for the PD-L1 positive rate suggest that this rate was not particularly high for non-small cell lung cancer and breast cancer^2^; therefore, we studied the impact of PTPRO on PD-L1 expression in hepatocellular carcinoma (HCC) monocytes and macrophages.

Antibodies to PD-L1/PD-1 have been approved for adjuvant therapy of HCC.^15^ Studies have also shown that the poor therapeutic effect of PD-1 adjuvant treatment is related to the increase of serum IL-6 concentration. IL-6 antibody, combined with PD-1/PD-L1 antibody treatment, has achieved significant results in animal tumor treatment model.^16^ Therefore, IL-6 may affect the adjuvant therapeutic effect of PD-1/PD-L1 through an unknown mechanism.

In the present study, we found a significant decrease in PTPRO expression in TAMs and peripheral monocytes in patients with HCC, and this decreased expression was related to increased PD-L1 expression. Relatively few studies have focused on PD-L1 expression by HCC monocytes, which are the cells of origin for TAMs; therefore, we investigated the mechanisms of signal transduction and transcription activation that control the downregulation of monocyte PTPRO and its contribution to PD-L1 expression.

## Methods

### Clinical samples

Blood or tissue samples were collected from 155 healthy volunteers and 165 patients with HCC who underwent surgical resection between January 2014 and October 2017 at The Affiliated Drum Tower Hospital of Nanjing University Medical School (Nanjing, Jiangsu, China). The blood samples were used for isolation of peripheral monocytes or tissue-infiltrating macrophages or for immunostaining. None of the patients had undergone anticancer therapy before surgery, and individuals with concurrent autoimmune disease, HIV infection, or syphilis were excluded. The patients’ clinical characteristics were classified according to the guidelines of the Union for International Cancer Control (TNM staging). All experiments were performed in compliance with government policies and the Helsinki Declaration. The individuals were informed about the study and gave consent prior to specimen collection. The clinical features of all involved patients in this study are presented in online supplementary table 1.

10.1136/jitc-2019-000285.supp1 Supplementary data

### Cell culture and reagents

Mouse (Hep1-6) HCC cell lines were maintained in Dulbecco’s modiﬁed Eagle’s medium (Invitrogen) supplemented with 10% fetal bovine serum (FBS; Gibco, USA). Human monocyte cell lines (U937, RRID:CVCL_0007 and THP-1, RRID:CVCL_0006) were maintained in RPMI 1640 medium (Invitrogen) supplemented with 10% FBS. All cell lines were purchased from the cell bank of the Chinese Academy of Sciences. All cell lines were tested for mycoplasma contamination prior to culture and use. All human cell lines have been authenticated using STR profiling.

For the macrophage stimulation, U937 and THP-1 cells were treated with 100 ng/mL phorbol myristate acetate (Sigma-Aldrich, St. Louis, MO, USA) for 2 days. The following inhibitors were purchased from Selleckchem: AZD 1480 (JAK2 inhibitor, S2162), 10058-F4 (c-MYC inhibitor, s7153), and bosutinib (c-Src inhibitor s1014). Human and mouse cytokines were purchased from Abcam (Cambridge, MA, USA), including human IL-6 (ab9627), mouse IL-6 (ab198572), human IFN-γ (ab119140), and mouse IFN-γ (ab9922). Human serum IL-6 levels were measured using a human IL-6 ELISA kit (ab46042; Abcam).

### Monocyte and macrophage isolation and in vitro stimulation

Human monocytes were obtained from healthy donors and patients with HCC by a two-step gradient centrifugation procedure using Ficoll and Percoll. Non-adherent cells were discarded, and the adherent cells were further isolated using human CD14 magnetic beads (130-050-201; Miltenyi Biotec, CA). The isolated cells were induced to form macrophages by incubation for 7 days in RPMI 1640 medium supplemented with 10% fetal calf serum and 100 ng/mL of recombinant human GM-CSF protein (ab88382; Abcam).

TAMs were isolated from liver cancer tissue specimens that were cut into small pieces and digested in collagenase IV (50 IU/mL). Dissociated cells were filtered through a 75 µm cell strainer and separated by Ficoll centrifugation, and the mononuclear cells were washed and resuspended in RPMI 1640 supplemented with 10% FBS (Gibco). The macrophages were subsequently isolated using human CD14 magnetic beads (130-050-201; Miltenyi Biotec).

Different genes and miRNAs were inserted into the lentiviral vector pLV (Clontech, CA, USA) to generate expression vectors. These expression vectors were mixed with lentiviral packaging Δ8.91 and envelope-expressing VSV-G plasmids to generate lentiviral particles in 293 T cells, as described previously.^17^ Viral particles were concentrated by ultracentrifugation and expression vector titers were determined. The isolated monocytes and macrophages were maintained in RPMI 1640 supplemented with 10% FBS (Gibco) for further analysis.

### RNA isolation and real-time PCR

Total RNA was isolated with TRIzol reagent. The cDNA was synthesized from total RNA using the iScript cDNA Synthesis Kit (1708890; Bio-Rad). The miR-25–3 p was detected by synthesizing its cDNA and measuring the transcription level in triplicate with the TaqMan microRNA assay kit (cat. no. 4427975; ThermoFisher, CA; ABI 7900; Life Technologies, CA, USA). Expression levels were calculated relative to U6. The primer pairs used in this study are listed in online supplementary table 2.

### Western blotting

Whole cell lysates were prepared as previously described.^18^ Equal amounts of protein were boiled, separated on 10% SDS-PAGE, transferred onto a PVDF membrane, and visualized using an enhanced chemiluminescence kit (Millipore, MA, USA). Antibodies against PTPRO (ab150834), STAT3 (Y705) (ab76315), STAT3 (S727) (ab30647), STAT3 (ab68153), STAT2 (Tyr690) (#88410; Cell Signaling), STAT2 (ab134192), STAT1 (Y701) (ab30645), STAT1 (ab31369), JAK2 (Y1007+Y1008) (ab32101), JAK2 (ab108596), c-MYC (s62) (ab51156), c-MYC (ab39688), human PD-L1 (ab205921), mouse PD-L1 (ab233482), and β-actin (ab6276) were purchased from Abcam. All uncropped western-blot figures are presented in online supplementary figures s13‒16.

### Immunofluorescence and immunohistochemical staining

Paraffin-embedded and formalin-fixed HCC samples were immunostained as previously described,^19^ using antibodies against human PD-L1 (ab205921), CD68 (ab955), CD3 (ab16669), TIM3 (ab242080), IL-1b (ab9722), p-JAK2 (Y1007+Y1008) (ab32101), p-c-SRC (Y418) (ab4816), STAT3 (ab68153), and p-c-myc (s62) (ab51156), followed by staining with Alexa-Fluor-488–conjugated anti-mouse IgG (1:500, ab150117), Alexa-Fluor-555–conjugated anti-rabbit IgG (1:1000, ab150074), and Alexa-Fluor-647–conjugated anti-rabbit IgG (1:500, ab150075) (Abcam) antibodies. Positive cells were detected by confocal microscopy (Zeiss, Oberkochen, Germany) and quantified using Image-Pro Plus software (Media Cybernetics, MD, USA).

### 3D coculture system

Macrophages isolated from wild-type (WT) or Ptpro KO mice were cocultured as a mixture with T cells (Cd3) isolated from WT mice and a mouse Hep1-6 HCC cell line at a ratio of 1:1:5 in a 3D petri dish (Micro-Tissues, RI, USA). The cells were treated differently and collected after 4 days. The macrophages and T cells were isolated from microtissues using Ficoll and further analyzed by flow cytometry and western blotting.

### Flow cytometry

Human peripheral monocytes and TAMs were isolated using anti-CD14 or anti-CD68 magnetic beads and stimulated with lipopolysaccharide (LPS) for 4 hours, followed by flow cytometry. The mouse macrophages were isolated as described previously^20^ and treated with LPS for 4 hours, followed by flow cytometry. Cells were stained with surface markers, fixed and permeabilized with IntraPre reagent (Beckman Coulter, Fullerton, CA), and finally stained with intracellular markers. Data were acquired on a FACSVantage SE instrument and analyzed with CellQuest software. The fluorochrome-conjugated antibodies used are described in online supplementary table 3.

### miRNA seq

Human peripheral monocytes were isolated using anti-CD14 magnetic bead from three patients with HCC and healthy controls, respectively. Small RNA library sequencing was performed on the Illumina Hiseq 2500/4000 by Gene Denovo Biotechnology (Guangzhou, China). The raw data were submitted to ArrayExpress (accession code E-MTAB-8998).

### Dual-luciferase reporter assay

The wide type and c-MYC binding site mutated promoter of miR-106b-25 cluster, 3′UTR of PTPRO containing the WT or mutant potential target site for miR-25–3 p was synthesized by Genescript (Nanjing, China) and inserted into the pGL4.10[luc2] vector (Promega, WI). For luciferase assays, stimulated U937 and THP-1 cells were co-transfected with pGL4-miR-25-PRO WT, and pGL4-miR-25-PRO MU, pGL4-PTPRO-WT 3′UTR, pGL4-PTPRO FL-WT 3′UTR or pGL4-PTPRO FL-MU 3′UTR, pGL4-PTPRO-MU 3′UTR, with miR-25–3 p mimics, or controls (GenePharma, China) using Lipofectamine 2000 (ThermoFisher, CA). Cells were harvested 48 hours after transfection and subjected to luciferase assays using a Dual-Luciferase Reporter Assay System (Promega, WI).

### In vivo model

An orthotopic mouse model of liver cancer was established as previously described, using Hep1-6 cells.^21^ One week before transplantation, adoptive cell transfer was performed, and this transfer was repeated once a week after orthotopic transplantation. A cell mixture containing 5×10^6^ macrophages transduced with lentiviral particles was transplanted into six WT mice via the tail vein. The tumor volume was calculated (length×width^2^×0.5). All mice were killed 5 weeks after transplantation, and organs were collected for further analysis.

### Statistical analysis

Data are presented as the mean±SEM. The χ^2^ test and Student’s t-test analysis of variance were used to evaluate differences in demographic and clinical characteristics. Kaplan-Meier survival curves were generated, and the log-rank test was used to investigate the significance of various variables for survival. All the expression experiments conducted in vitro were repeated at least three times with triplicate samples. Pearson’s correlation analysis was used to analyze the relationship of associated factors. Statistical analysis was performed using STATA V.9.2 and presented with the GraphPad Prism (CA, USA). In all cases, p value <0.05 was considered significant.

## Results

### PTPRO is negatively associated with the PD-L1 expression by monocytes from human HCC

**Figure 1:**
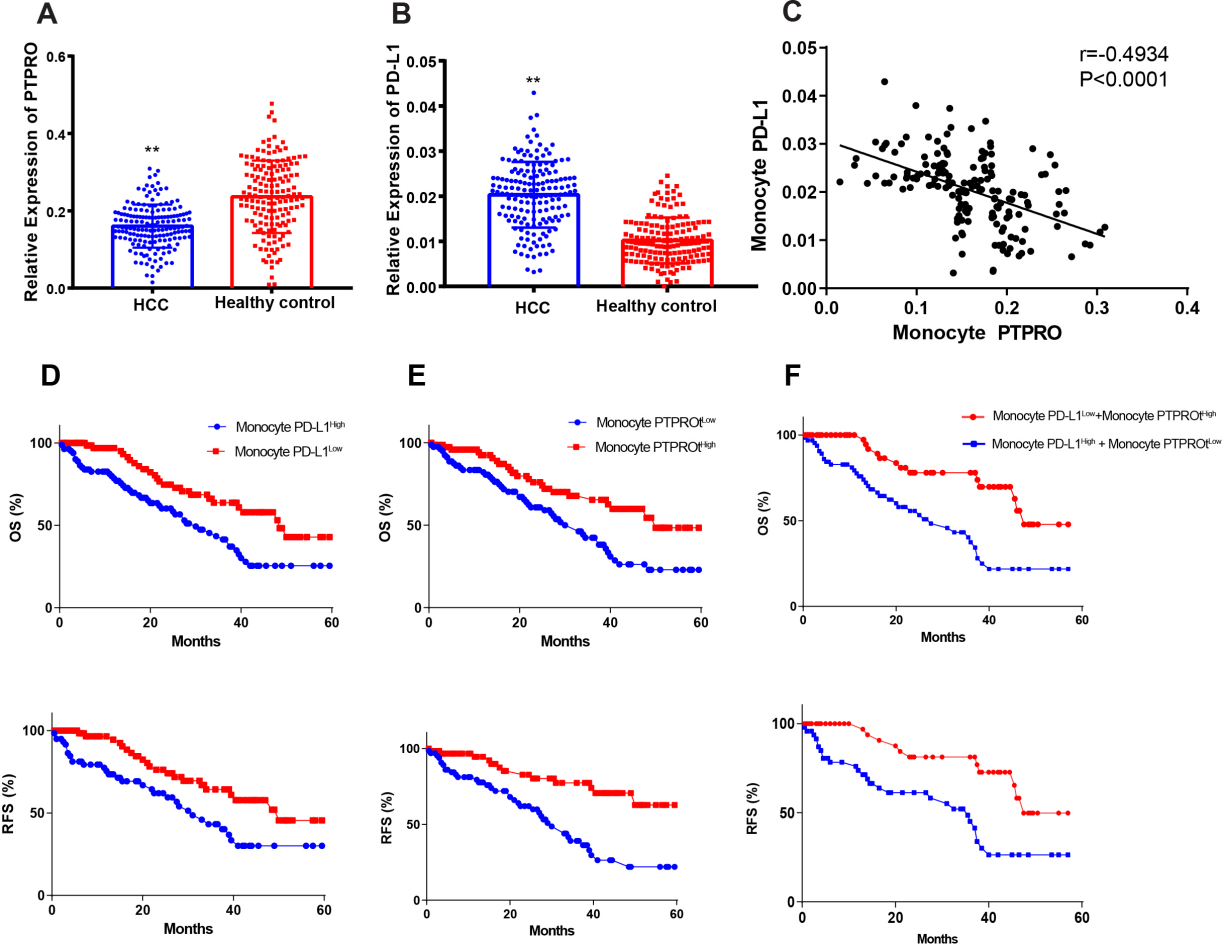
Protein tyrosine phosphatase, receptor type O (PTPRO) expression is negatively associated with PD-L1 expression in monocytes and served as indices for prognosis of patients with hepatocellular carcinoma (HCC). Human monocytes were isolated using anti-CD14 magnetic beads from 165 patients with HCC and 155 healthy controls. Transcription of PTPRO (A) and PD-L1 (B) were detected using real-time PCR. Data are presented as means±SEM and were analyzed with Student’s t-test (^**^p<0.01). (C) Linear correlation studies were performed for PTPRO vs PD-L1 in monocytes of patients with HCC. Each experiment was performed in triplicate. Kaplan-Meier survival curves were generated, and the log-rank test was used to investigate the significance of various variables for overall survival (OS) and relapse-free survival (RFS) due to different PTPRO and PD-L1 expressions in HCC monocytes. Detailed analyses: (D) monocyte PD-L1 vs OS, monocyte PD-L1 vs RFS; (E) monocyte PTPRO vs OS, monocyte PTPRO vs RFS; (F) multiple-variant survival analysis consisted of monocyte PTPRO and PD-L1 vs OS; multiple-variant survival analysis consisted of monocyte PTPRO and PD-L1 vs RFS.

We studied the expression of PTPRO and PD-L1 expression in monocytes isolated from the peripheral blood using magnetic beads labeled with anti-CD14 antibody. Significantly decreased expression PTPRO but increased PD-L1 transcription levels revealed in monocytes from patients with HCC when compared with monocytes from control subjects (figure 1A, B). Linear correlation analysis revealed negative correlations between PTPRO expression and PD-L1 in human HCC monocytes (PTPRO vs PD-L1, r=−0.4934, p<0.0001; figure 1C). We also found that the level of monocyte PTPRO was significantly associated with patient’s age, hepatitis B virus infection, tumor size and tumor number (online supplementary table 1).

### Monocyte PTPRO and PD-L1 expressions serve as indicators for prognosis of post-surgery patients with HCC

We had follow-up data for the 5-year survival for 129 of the 165 patients with HCC. The patients with HCC were subdivided into high and low expression subgroups according to the gene transcription medians of PD-L1 and PTPRO in the monocytes. Kaplan-Meier curves revealed that patients with either PD-L1^High^ or PTPRO^Low^ had significantly lower post-surgery overall survival (OS) and relapse-free survival (RFS) when compared with PD-L1^Low^ or PTPRO^High^ patients (figure 1D, E) (Monocyte PD-L1^High^ vs Monocyte PD-L1^Low^: p=0.0018, HR 2.144, 95% CI 1.341 to 3.426 for OS; p=0.0069, HR 2.099, 95% CI 1.209 to 3.645 for RFS; Monocyte PTPRO^High^ vs Monocyte PTPRO^Low^: p=0.0017, HR 2.162, 95% CI 1.348 to 3.467 for OS; p<0.0001, HR 3.332, 95% CI 1.911 to 5.811 for RFS, by the log-rank test). Multiple-variant analysis revealed that Monocyte PD-L1^Low^+Monocyte PTPRO^High^ patients had significantly higher OS and RFS compared with Monocyte PD-L1^High^+Monocyte PTPRO^Low^ patients (figure 1F) (Monocyte PD-L1^Low^+Monocyte PTPRO^High^ vs Monocyte PD-L1^High^+Monocyte PTPRO^Low^ p=0.0002, HR 3.019, 95% CI 1.348 to 1.775 for OS; and p=0.0010, HR 3.018, 95% CI 1.592 to 5.719 for RFS). These findings indicated that monocyte PTPRO and PD-L1 expressions are valuable indicators for predicting the prognosis of patients with HCC. However, the underlying mechanism requires further exploration.

### Decreased expression of PTPRO in TAMs is related to increased T-cell exhaustion within the HCC TME

**Figure 2:**
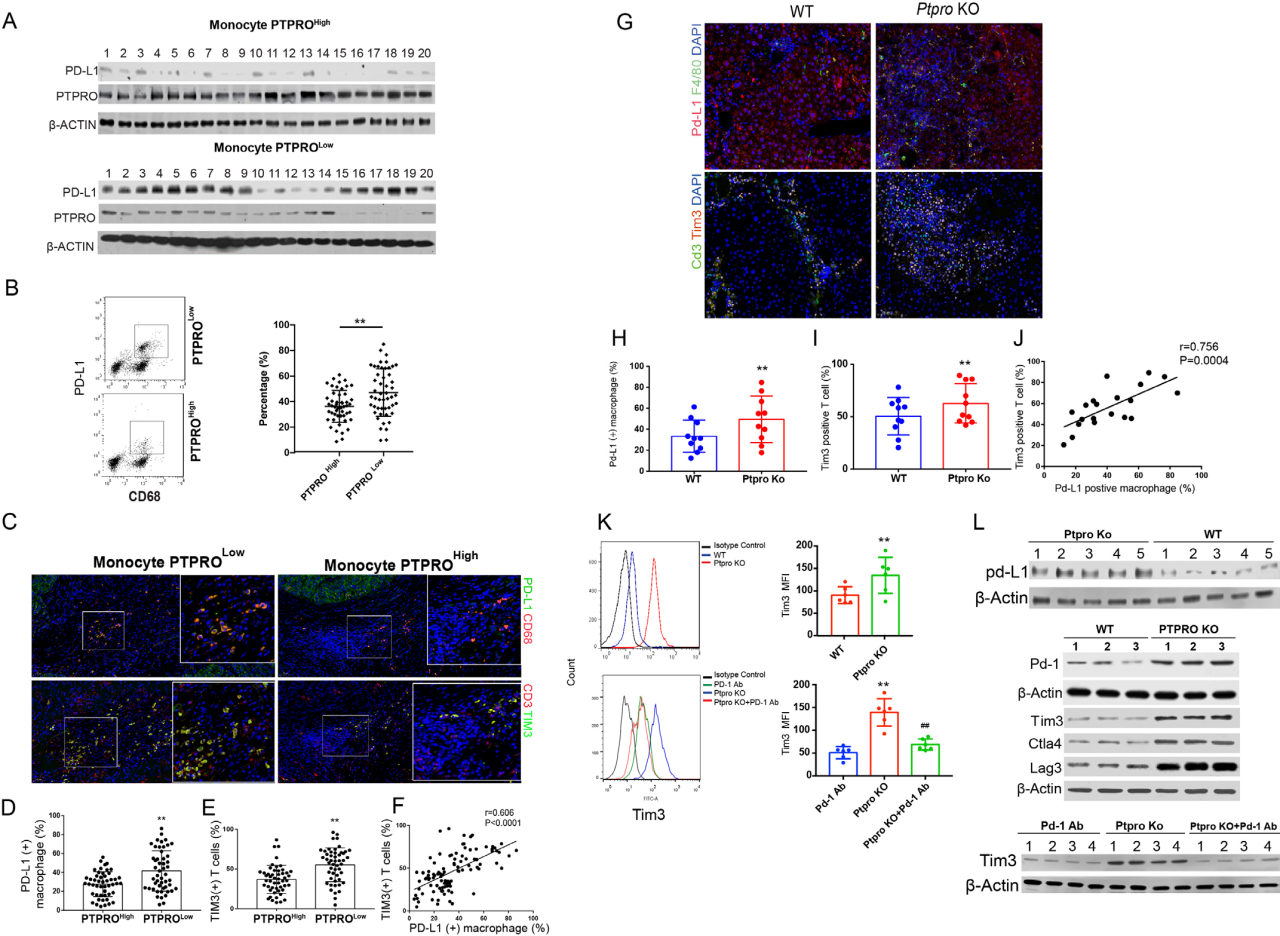
Decreased expression of protein tyrosine phosphatase, receptor type O (PTPRO) in tumor-infiltrating macrophages was related to increased T-cell exhaustion within the hepatocellular carcinoma (HCC) tumor microenvironment (TME). Tumor-infiltrating macrophages were isolated from patients with HCC and divided into two subgroups according to the monocyte PTPRO expression. PD-L1 and PTPRO expression was detected by western blotting (n=20 in each group) (A). PD-L1 expression was analyzed by flow cytometry (n=50 in each group) (B). (C, upper panel) Representative figure showing immunofluorescence staining for PD-L1/CD68 and CD3/TIM3 in human HCC tissues from the PTPRO^Low^ and PTPRO^High^ monocyte groups. A comparison of the percentages of PD-L1 (+) macrophages and Tim3 (+) T cells in the two groups is presented in (D) and (E) (n=50 for each group). (F) Linear correlation between the percentages of PD-L1 (+) macrophages and Tim3 (+) T cells (n=100). (G) Representative figure of immunofluorescence staining for Pd-L1/F4/80 and Cd3/Tim3 in mouse DEN-induced HCC tissues from wild-type (WT) and *Ptpro* KO mice. A comparison of the percentages of Pd-L1 (+) macrophages and Tim3 (+) T cells in the two groups is presented in (H) and (I) (n=9 for each group). (J) Linear correlation between the percentages of Pd-L1 (+) macrophages and Tim3 (+) T cells (n=18). (K) Flow cytometry analysis of Tim3 expression in T cells (gated by CD3); Tim3-positive cells were compared within different treatments. ^**^p<0.01, compared with Pd-1 Antibody (Pd-1 Ab) control, ^##^p<0.01, compared with Ptpro KO macrophage group. (L) Analysis of the expression of indices indicated in the figures in Ficoll-isolated 3D cultured cells by western blotting. Each experiment was performed in triplicate. Data are presented as the mean±SEM and were analyzed by Student’s t-test (^**^p<0.01).

The PD-L1 expression in HCC-infiltrating macrophages of the PTPRO^Low^ and PTPRO^High^ groups (n=20 for each group) was examined in macrophages extracted from fresh tumor tissues and further purified with CD 68 magnetic beads. We first used western blotting to compare the expression of PD-L1 in the macrophages from the PTPRO^Low^ and PTPRO^High^ expression groups (n=20), and we found a significantly higher expression of PD-L1 in the PTPRO^Low^ group (figure 2A). The expressions of PD-L1 in the macrophages were also detected by flow cytometry following stimulation with brefeldin A (10 µg/mL; 5 hours at 37°C) (n=50 for each group). The results were similar to those obtained with western blotting, indicating a higher number of PD-L1–positive macrophages (CD68) in the PTPRO^Low^ group than in the PTPRO^High^ group (figure 2B).

We then investigated the relationship between PD-L1–expressing macrophages and exhausted tumor-infiltrating T cells (TILs) (n=50 for each group). Significantly more PD-L1–positive macrophages were detected in the tumor tissues of the PTPRO^Low^ patients, and this was positively related to significantly more Tim3-positive (one of the markers for T-cell exhaustion) TILs (r=0.606, p<0.0001) (figure 2C–F). These results suggested an association between monocyte/macrophage PTPRO expression and immunosuppression in human HCC.

### PD-L1 expression was increased in TAMs and was associated with exhausted T cells in HCC of PTPRO KO mice

We have reported that PTPRO can suppress hepatocarcinogenesis in mouse hepatocytes via suppression of Jak2/Stat3 and c-Src/Stat3 activation.^14^ We also previously reported that deletion of PTPRO truncated (PTPROt) decreased the quantity and quality of cytotoxic T lymphocytes (CTLs) in a mouse HCC model.^22^ Therefore, we doubted that the deletion of PTPRO in macrophages would suppress CTLs via PD-L1–induced T-cell exhaustion. We therefore analyzed PD-L1 expression in mouse HCC tissues in the present study, and we found a significant enhancement of PD-L1 in HCC tissues in PTPRO KO mice when compared with WT control, and especially in TAMs (figure 2G, H). We also found a significant increase in the percentage of exhausted T cells (Tim3 positive) in HCC tissues from PTPRO KO mice when compared with WT controls (figure 2G‒I). Moreover, the percentage of exhausted T cells was positively correlated to the percentage of Pd-L1 positive macrophage (r=0.756, p=0.0004) (figure 2J). We excluded the effects caused by varied PD-L1 expression due to different HCC tissues by constructing a 3D culture system that included the mouse Hep1-6 HCC cell line, macrophages isolated from PTPRO KO and WT control mice, and T cells isolated from WT mice. We found that deletion of PTPRO in the macrophages could increase T-cell exhaustion by enhanced production of PD-L1, and that the T-cell exhaustion could be significantly restored by treatment with PD-1 antibody (figure 2K, L). We found that decreases in PTPRO could promote PD-L1 expression in macrophages/monocytes and induce T-cell exhaustion in both human and mouse HCC.

### PTPRO suppresses PD-L1 expression via JAK2/STAT1 and JAK2/STAT3/c-MYC activation in HCC macrophages or monocytes

**Figure 3:**
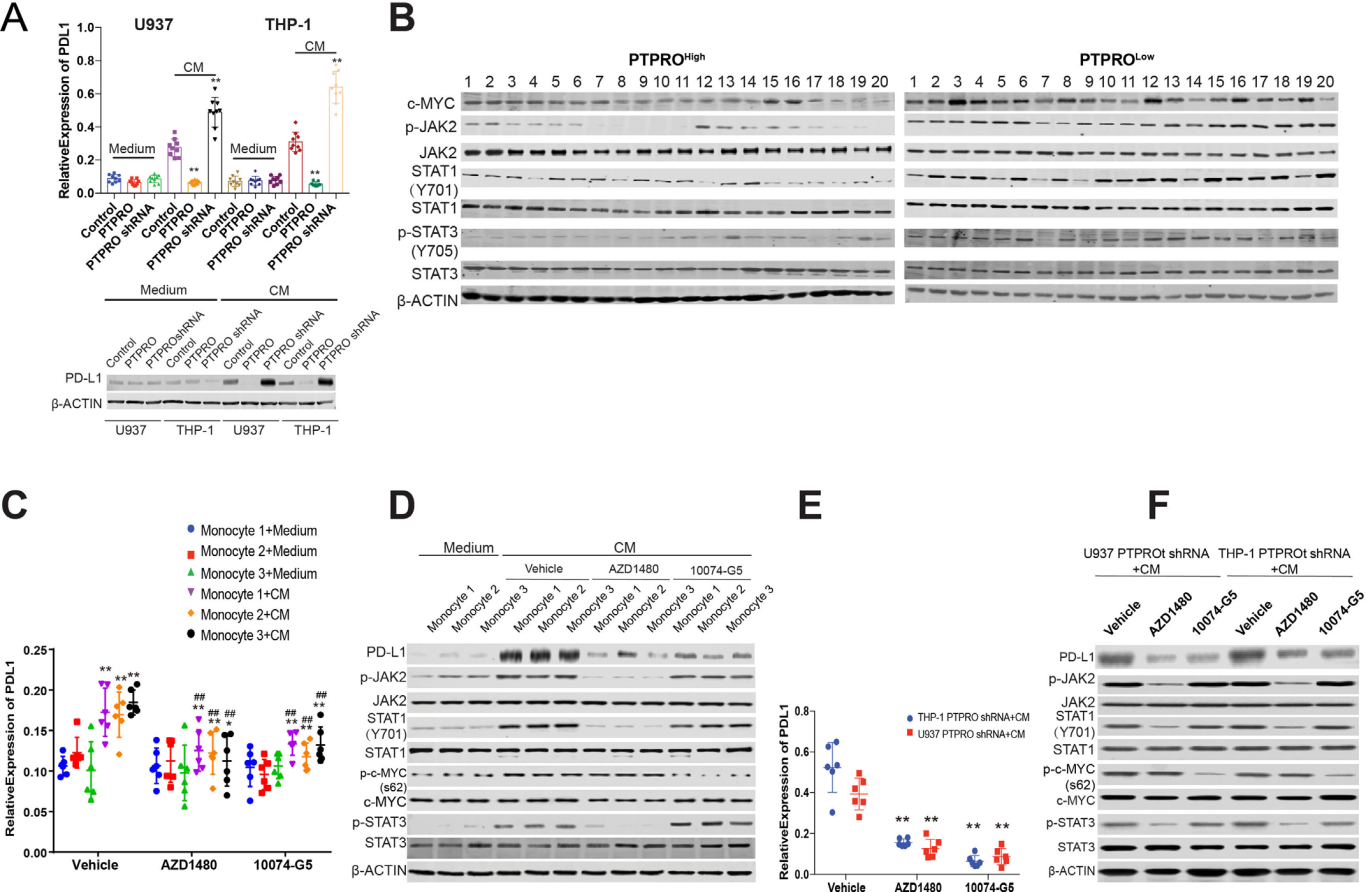
Protein tyrosine phosphatase, receptor type O (PTPRO) expression by macrophages or monocytes suppresses PD-L1 expression via STAT3/c-MYC activation in hepatocellular carcinoma (HCC). (A) U937-derived and THP-1–derived macrophages with different PTPRO expression levels were treated with control medium (Medium) and tumor conditional medium (CM), and the transcription (upper panel) and protein expression (lower panel) of PD-L1 were detected by real-time PCR and western blotting, respectively. (B) Tumor-infiltrating macrophages were isolated from patients with HCC and divided into two subgroups according to monocyte PTPRO expression. The expression of c-MYC, p-STAT3(Y705), and total STAT3 was detected by western blotting (n=20 in each group). (C) Human monocyte-derived macrophages were treated with control medium (Medium) and tumor conditional medium (CM), and the CM-treated macrophages were further treated with vehicle control, AZD1480, or 10074-G5. The transcription of PD-L1 was determined by real-time PCR. (D) Human monocyte-derived macrophages were treated as in (C), ^**^p<0.01, compared with the Medium control, ^##^p<0.01, compared with the vehicle control with same treatment, and the protein expression of PD-L1 p-c-MYC, total c-MYC, p-STAT3 (Y705), and total STAT3 was detected by western blotting. (E) THP-1 and U937 treated with PTPRO shRNA were treated with tumor conditional medium (CM), and further treated with vehicle control, AZD1480, or 10074. PDL-1 transcription was then determined by real-time PCR. (F) THP-1 and U937 PTPRO shRNA cells were treated as described in (E), and the protein expression of PD-L1 p-c-MYC, total c-MYC, p-STAT3 (Y705), and total STAT3 were detected by western blotting. Each experiment was performed in triplicate. Data are presented as the mean±SEM and were analyzed by Student’s t-test (^**^p<0.01).

We explored the molecular mechanism underlying PTPRO effects by overexpression and knockdown of PTPRO in THP-1–derived and U937-derived macrophages, respectively (online supplementary figure s1), and following further treatment with or without tumor conditioned medium (CM). The expression of PD-L1 significantly decreased in the PTPRO-overexpressing THP-1 and U937 cells but significantly increased in PTPRO KO cells. However, the PD-L1 expression did not differ unless the cells were stimulated with CM (figure 3A), indicating that PTPRO can suppress in vitro PD-L1 expression in macrophages with certain stimulations.

Our previous studies indicated that PTPRO suppresses JAK2 activation in HCC cells, and other research has also indicated that activation of c-MYC, a downstream regulator of STAT3,^23^ can enhance PD-L1 expression.^24^ Therefore, we postulated that PTPRO might suppress PD-L1 expression by suppression of both the JAK2/STAT1 and JAK2/STAT3/c-MYC signaling pathways. We tested this hypothesis by evaluating STAT1 and STAT3 activation in TAMs from patients with HCC. We observed a stronger activation of JAK2/STAT1 (Y701) and JAK2/STAT3 (Y705), as well as increased c-MYC expression, in the PTPRO^Low^ group than in the PTPRO^High^ group (n=20 for each group) (figure 3B). Taken together with the PD-L1 expression shown in figure 2A, these data suggested that PD-L1 expression might be associated with JAK2/STAT1 and JAK2/STAT3/c-MYC activation in human HCC in monocyte or TAM.

We investigated the potential mechanism of PTPRO suppression on PD-L-1 using inhibitors that specifically targeted JAK2 (AZD1480) and c-MYC (10074-G5). Suppression of JAK2 and c-MYC decreased the PD-L1 transcription and expression. Culture of monocytes isolated from healthy controls (n=3) in CM increased the transcription and expression of PD-L1 and further increases were obtained by inhibiting JAK2 and c-MYC. Blockade of JAK2 signaling, in particular, decreased PD-L1 expression to levels similar to those observed in the medium control (figure 3C, D). As was observed in human macrophages, PD-L1 expression also significantly decreased when JAK2 and c-MYC were blocked in THP-1-PTPRO shRNA and U-937-PTPRO shRNA cells; this effect was particularly evident following JAK2 suppression (figure 3E, F).

Jak2/Stat1 and Jak2/Stat3/c-Myc signaling was also enhanced in macrophages from PTPRO KO mice when compared with WT control mice (online supplementary figure s2). We examined the regulation of JAK2 by PTPRO in macrophages by analyzing the relative distribution of PTPRO and JAK2. PTPRO was found predominantly on the cell membrane, whereas JAK2 was co-localized with PTPRO in U937 cells, which indicated a possible binding of PTPRO to JAK2 (online supplementary figure s3). Co-immunoprecipitation (co-IP) assays revealed increased levels of JAK2 in the PTPRO co-IP assays over control levels following treatment with IL-6, which indicated that PTPRO could regulate JAK2 by direct binding (online supplementary figure s4). These findings indicated that PTPRO can suppress PD-L1 expression in monocytes and macrophages by suppressing JAK2/STAT1 and JAK2/STAT3/c-MYC. Based on a previous report,^13^ we also performed a luciferase gene reporter assay, using PD-L1 promoter (−500 bp), which contained binding site of STAT1/STAT3 and STAT2/STAT5. We found that INF-γ can increase the PD-L1 promoter significantly and deletion of STAT1/STAT3 binding site but not STAT2/STAT5 binding site can decrease PD-L1 promoter dramatically; this result might suggest that STAT1 and STAT3 were dominant in PD-L1 transcription in macrophage (online supplementary figure s5).

### IL-6 promotes PD-L1 expression in macrophages by deregulating PTPRO both directly and via an IFN-γ–dependent mechanism

**Figure 4:**
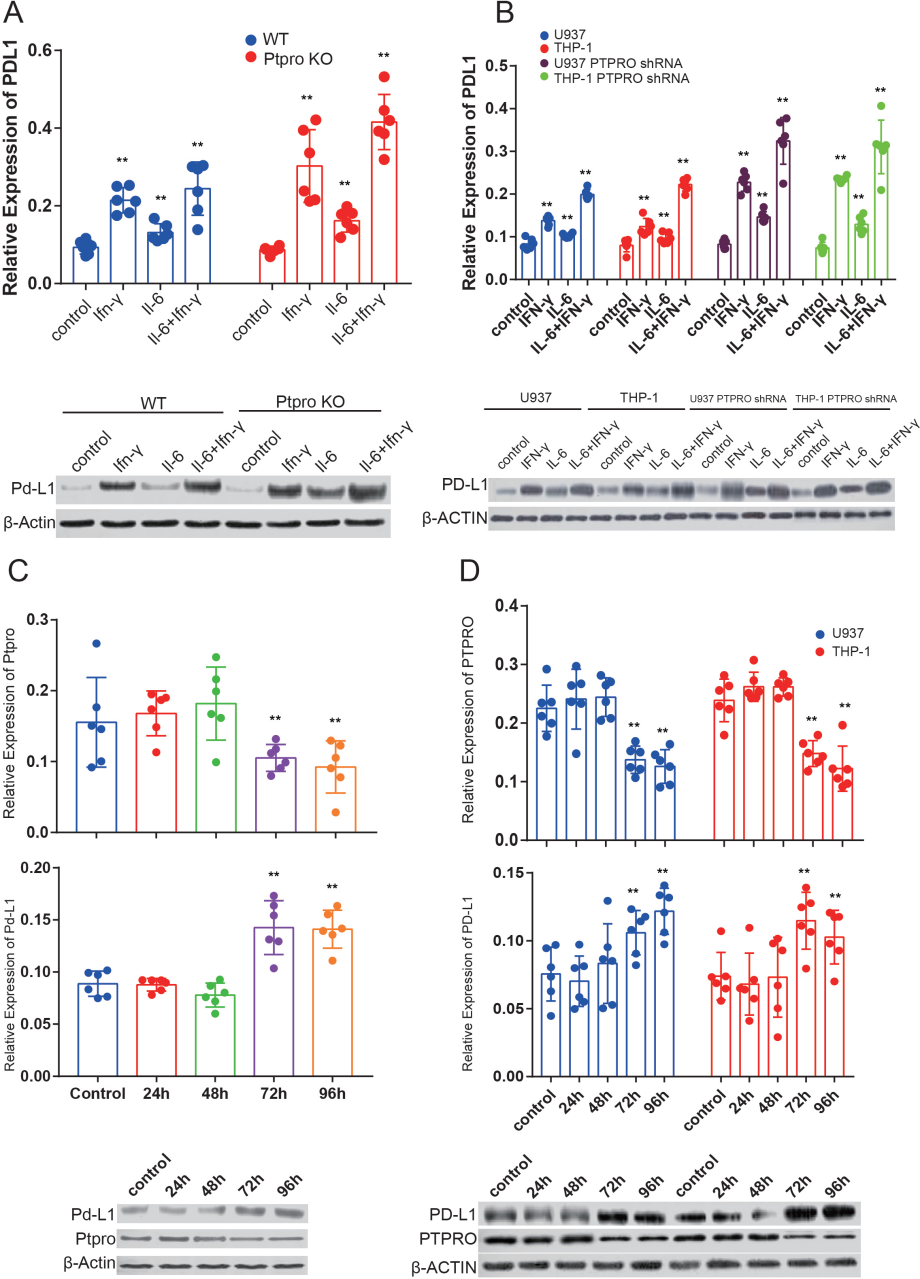
IL-6 promotes PD-L1 expression in macrophage both directly and IFN-γ dependently by deregulating protein tyrosine phosphatase, receptor type O (PTPRO). (A) Macrophages isolated from wild-type (WT) and PTPRO KO mice were treated with IFN-γ (50 ng/mL), IL-6 (50 ng/mL), and IFN-γ+IL-6 (50 ng/mL) for 72 hours and the transcription and protein expression of Pd-L1 were determined by using real-time PCR and western blotting, respectively. (B) U937-, THP-1–, U937 PTPRO shRNA–, and THP-1 PTPRO shRNA–derived macrophages were treated with IFN-γ (50 ng/mL), IL-6 (50 ng/mL), or IFN-γ+IL-6 (50 ng/mL each), and the transcription and protein expression of PD-L1 were determined by real-time PCR and western blotting, respectively. (C) Macrophages isolated from WT and PTPRO KO mice were treated with IL-6, and the expression of PD-L1 and PTPRO was detected by real-time PCR and western blotting at different times after IL-6 treatment. (D) U937-, THP-1–, U937 PTPRO shRNA–, and THP-1 PTPRO shRNA–derived macrophages were treated with IL-6, and the expression of PD-L1 and PTPRO was detected by real-time PCR and western blotting at different times after IL-6 treatment. Each experiment was performed in triplicate. Data are presented as the mean±SEM and were analyzed by Student’s t-test (^**^p<0.01).

No significant increase was observed in PD-L1 expression in the monocytes/macrophages in the absence of CM (figure 3A). We used a cytokine array to find the difference in cytokine secretion between control and tumor conditional medium treated macrophages, and the result indicated that IL-6 and INF-γ were the most variable of all cytokines (online supplementary figure s6). Based on previous knowledge,^6 24–26^ we postulated that IFN-γ and IL-6 might be the activators for JAK2/STAT1/PD-L1 and JAK2/STAT3/c-MYC/PD-L1 activation in the monocytes/macrophages. We tested this hypothesis by treating the macrophages with IL-6 (50 ng/mL) and IFN-γ (50 ng/mL), and we found that both INF-γ and IL-6 increased the Pd-L1/PD-L1 expression in *Ptpro* KO and PTPRO knockdown macrophages. IFN-γ, but not IL-6, also increased PD-L1 expression in WT and normal-PTPRO–expressing macrophages 24 hours after treatment (online supplementary figure s7 A‒D). IL-6 treatment significantly increased Pd-L1/PD-L1 expression both directly and in an INF-γ–dependent manner in WT and normal-PTPRO–expressing macrophages 72 hours after the treatment. The IL-6 promotion was particularly evident for IFN-γ–induced Pd-1/PD-L1 expression in *Ptpro* KO and PTPRO knockdown macrophages (figure 4A, B). This result could indicate that PTPRO expression level is essential for IL-6–induced Pd-L1/PD-L1 expression in macrophages.

We also treated WT mouse macrophages and U-937–derived and THP-1–derived macrophages with IL-6 and tracked the expression of PTPRO at different times. The IL-6 treatment decreased the transcription and expression of PTPRO in both mouse and human macrophages 72 hours after treatment (figure 4C, D and online supplementary figure s8). Monitoring of PD-L1 also showed that its expression paralleled the PTPRO expression both in human and mouse macrophage (figure 4C, D). Therefore, IL-6 activated the STAT3/c-MYC/PDL-1 axis and deregulated PTPRO action in monocytes/macrophages.

### IL-6 downregulates PTPRO in HCC monocytes by up-regulating miR-25–3 p by STAT3/c-MYC signaling

**Figure 5:**
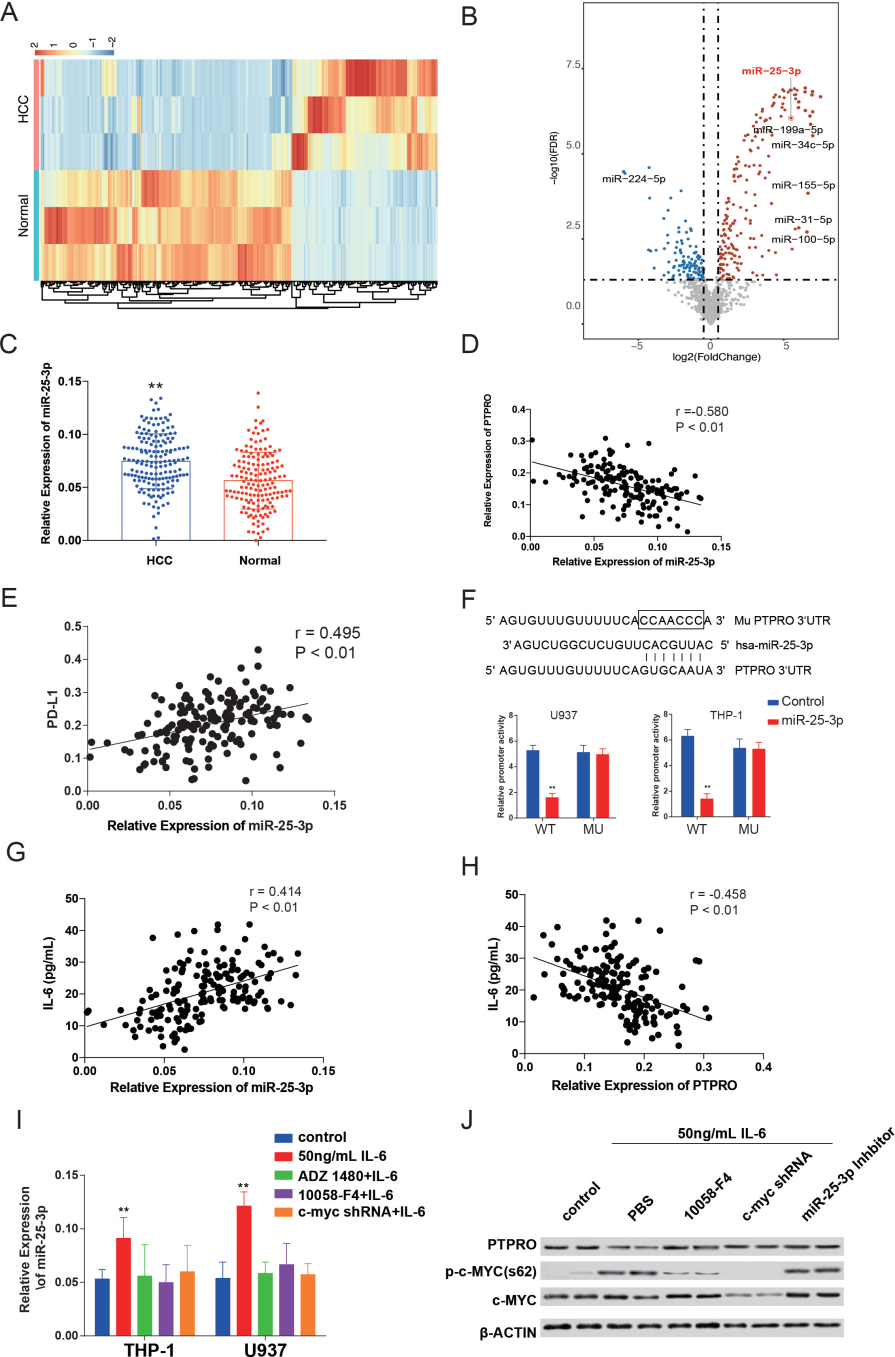
IL-6 downregulates protein tyrosine phosphatase, receptor type O (PTPRO) in hepatocellular carcinoma (HCC) monocytes by upregulating miR-25–3 p via STAT3/c-MYC. (A, B) Heatmap and volcano plot indicating significantly expressed miRNA within monocytes in patients with HCC compared with the healthy controls. (C) Anti-CD14 magnetic beads were used to isolate human monocytes from 165 patients with HCC and 155 healthy controls. Transcription of miR-25–3 p was detected using real-time PCR and compared between the two groups. ^**^p<0.01, Student’s t-test. (D, E) The linear correlations between expression of miR-25–3 p and PTPRO or PD-L1 were analyzed in HCC monocytes. (F) The potential binding site and mutation in the 3′ untranslated region (UTR) are indicated in the schematic figure. A luciferase reporter gene assay was carried out to test the promoter activity of PTPRO regulated by miR-25–3 p on the 3′UTR of PTPRO. (G, H) Linear correlations between serum IL-6 and monocyte PTPRO, and serum IL-6 and monocyte miR-25–3 p were analyzed in patients with HCC. (I) THP-1–derived and U937-derived macrophages were treated as indicated, and expression of miR-25–3 p was determined by real-time PCR. ^**^p<0.01, Student’s t-test, compared with control group. (J) Cell signaling was analyzed by western blotting. Each experiment was performed in triplicate. Data are presented as the mean±SEM and were analyzed with Student’s t-test (^**^p<0.01).

We also compared the miRNA expression in the monocytes between patients with HCC and the healthy controls, and the results indicated 313 miRNAs significantly upregulated and 114 miRNAs significantly downregulated in HCC monocytes (figure 5A, B). In addition, we also queried potential binding miRNAs within the 3′UTR of PTPRO (online supplementary figure s9); among these 313 upregulated miRNAs, we found that only miR-25–3 p had the potential to bind to the 3′UTR of PTPRO. In addition, serum IL-6 levels were significantly increased in most of the patients with HCC.^27^ These findings led us to question whether links might exist between increased serum IL-6 levels and decreased PTPRO expression in monocytes or macrophages. We also queried whether these miRNAs might be the linkers.

Expression of miR-25–3 p was confirmed in human monocytes, and its expression was significantly higher in the HCC monocytes than in the healthy control monocytes (figure 5C). We examined the potential relationship between miR-25–3 p and PTPRO expression by linear correlation studies of the expression of miR-25–3 p and PTPRO or PD-L1 in monocytes. The miR-25–3 p expression in HCC monocytes was negatively correlated with PTPRO expression (r=−0.580, p<0.01) (figure 5D) and positively correlated with PD-L1 expression (r=0.495, p<0.01) (figure 5E) in human HCC monocytes.

We speculated that miR-25–3 p could regulate PTPRO expression in monocytes by targeting the 3′UTR. The miRNA 3′UTR prediction results indicated that miR-25–3 p can align with 1381–1388 bp at the 3′UTR of PTPRO (figure 5F). We verified this regulation by generating mutated PTPRO 3′UTR and sub-cloning it into the pGL4-Luc vector. Transfection of U973-derived and THP-1–derived macrophages with the mutation in the 3′UTR of PTPRO significantly decreased the transcription activity in both cell types, thereby confirming that miR-25–3 p regulated PTPRO expression (figure 5F). Moreover, we confirmed that miR-25–3 p can decrease both transcription and protein expression by targeting the 3′UTR of PTPRO in macrophages (online supplementary figure s10).

Previous studies have reported that c-MYC can regulate the miR-25–3 p cluster.^28^ Also, we further confirmed such regulation by using a luciferase gene reporter assay (online supplementary figure s11). We surmised that upregulation of IL-6 in HCC monocytes and macrophages might promote the expression of miR-25–3 p via activation of the STAT3/c-MYC signaling pathway. We verified this potential regulation mechanism by investigating the serum IL-6 levels in patients with HCC by ELISA (online supplementary figure s12) and by performing a linear correlation study between the serum IL-6 levels and expression of miR-25–3 p or PTPRO in HCC monocytes. We found a clear positive linear correlation between serum IL-6 and monocyte expression of miR-25–3 p or PTPRO (IL-6 vs miR-25–3 p, r=0.414, p<0.01; IL-6 vs PTPRO, r=−0.458, p<0.01) (figure 5G, H). We then treated THP-1–derived and U937-derived macrophages with 50 ng/mL IL-6 and found that miR-25–3 p increased significantly 48 hours after the treatment and that miR-25–3 p transcription was restored using a c-MYC inhibitor (10058-F4) or by treatment with c-MYC shRNA (figure 5I). This signaling pathway analysis also indicated that the decreased expression of PTPRO by IL-6 was accompanied by an increased activation of STAT3/c-MYC signaling 72 hours after treatment. Treatment of U937-derived and THP-1–derived macrophages with AZD 1480, 10058-F4, c-MYC shRNA, or an miR-25–3 p inhibitor restored the expression of PTPRO that had been reduced by IL-6 treatment (figure 5J).

### c-MYC/miR-25–3 p/PTPRO axis promotes in vivo PDL-1 expression and tumor growth by targeting monocyte PTPRO

**Figure 6:**
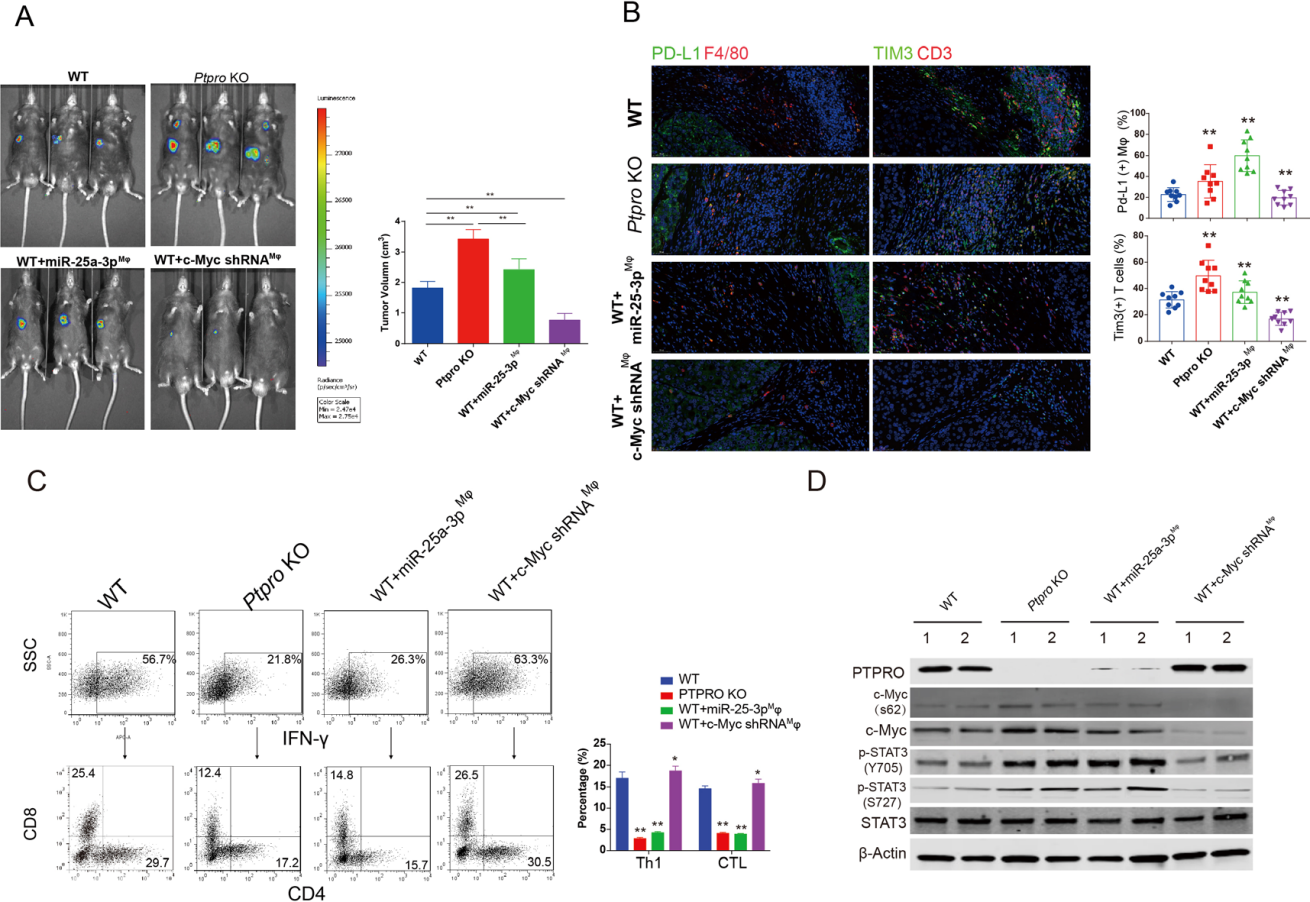
c-MYC/miR-25–3 p/PTPRO axis promotes in vivo PDL-1 expression and tumor growth by targeting monocyte protein tyrosine phosphatase, receptor type O (PTPRO). (A) An orthotopic tumor transplantation model with adoptive cell transfer was created as indicated. Tumor volumes were compared between the groups. (B) Representative figures of immunofluorescence staining for Pd-L1/F4/80 and Cd3/Tim3 are presented in left panel for tumors obtained from the different mouse groups indicated in the figure. Comparisons of the percentages of Pd-L1 (+) macrophages and Tim3 (+) T cells in the four groups are presented in the right panel. (C) Tumor-infiltrating monocytes were isolated, and the percentages of Th1 cells and CTLs were determined by flow cytometry. (D) Activation of STAT3/c-MYC signaling in tumor-infiltrating macrophages was determined by western blotting. Each experiment was performed in triplicate. Data are presented as the mean±SEM and were analyzed with Student’s t-test (^**^p<0.01).

We investigated TAM differentiation and the tumor growth–promoting effects of the c-MYC/miR-25–3 p/PTPRO axis using an orthotopic tumor transplantation model with adoptive cell transfer. As in our previous studies,^22^ we saw a significant increase in tumor volume in *Ptpro* KO mice when compared with the WT controls. Adoptive transfer of miR-25–3 p–overexpressing macrophages into WT mice (WT+miR-25–3 p^Mφ^) significantly increased the tumor volume, though this volume was still smaller than the volume of the tumors that developed in the *Ptpro* KO mice. WT mice that underwent transfer of c-Myc-knockdown macrophages (WT+c Myc shRNA^Mφ^) showed a dramatic decrease in tumor volume (figure 6A).

The TME was investigated by immunofluorescence staining of the Pd-L1 positive macrophages and exhausted T cells within the tumor. The percentage of Pd-L1 (+) macrophages and Tim3 (+) T cells increased in *Ptpro* KO and WT mice following adoptive transfer of miR-25–3 p–overexpressing macrophages. Conversely, the percentage of Pd-L1 (+) macrophages and Tim3 (+) T cells decreased significantly following transfer of c-MYC–silenced macrophages into WT mice (figure 6B). The percentage of Th1 cells and CTLs within the tumor decreased significantly with the adoptive transfer of miR-25–3 p–overexpressing macrophages into WT mice. Conversely, the percentage of Th1 cells and CTLs increased significantly following transfer of c-MYC–silenced macrophages into WT mice (figure 6C).

We also investigated the signaling pathway in TAMs. Activation of STAT3 was significantly increased following depletion of *Ptpro*, but STAT3 activation increased significantly in WT mice following adoptive transfer of miR-25–3 p–overexpressing macrophages (figure 6D).

## Discussion

IL-6 is a classic proinflammatory cytokine that is secreted by both T cells and macrophages. IL-6 is now regarded as a key cytokine linking inflammation to tumorigenesis in HCC, gastric, and lung cancers, as well as leukemia.^29 30^ These malignancies are associated with significant increases in the serum concentration of IL-6, making this cytokine an ideal biomarker for prognostic prediction and diagnosis.^31–33^ Sustained upregulation of IL-6 can promote tumors by activation of JAK2/STAT3 signaling within the tumor or in premalignant cells.^29^ A study published in 2007 indicated that IL-6 is an essential cytokine that links inflammation and gender disparity in tumorigenesis.^27^ IL-6 also plays an oncogenic role in obesity-linked HCC.^34^ All this evidence implies that IL-6 might be a valuable target for future cancer therapy, especially for tumors associated with an immunosuppressive TME. However, the results from clinical trials using an IL-6 antibody (siltuximab) have been disappointing. One of the main explanations for the poor clinical response was immunosuppression induced by PD-1. A recent study revealed an increase in IL-6 in patients treated with nivolumab (a PD-1 antibody), and evidence from a mouse model indicated that a combined blockade of both PD1/PD-L1 and IL-6 could significantly increase the therapeutic effects.^16^ In the present study, we found that a sustained increase in serum IL-6 impaired the anti-inflammatory activity of monocytes, contributed to the potential immunosuppression caused by an increased percentage of PD-L1–producing TAMs within the tumor, and decreased expression of PTPRO within the monocytes. Based on the immunofluorescence staining of liver cancer tissues, we found that most of the PD-L1–producing TAMs were located in the edge of tumor. We speculated that the accumulation of PD-L1–producing TAMs can block the infiltration of CD8 cells, which are the most predictive indices for immunotherapy.^35^

PTPRO is a member of the R3 subtype family of receptor-type protein tyrosine phosphatases. JAK/STAT signaling can be inactivated by dephosphorylation of tyrosine.^14^ In the present study, we reported that decreased expression of PTPRO was linked to increasing immunosuppression caused by upregulation of PD-L1 through the deregulation of JAK2/STAT1 and JAK2/STAT3 activation. This finding agreed with many previous studies indicating that PTPRO can suppress tumorigenesis or immunosuppression by inhibiting JAK/STAT signaling.^14 22 29^ Interestingly, Sung Hee Kil *et al* reported that PD-L1 is regulated in lymphoma by both INF-γ and IL-6 through STAT1 and STAT3 signaling.^36^ Some of these results were similar to ours, including upregulation of PD-L1 expression by IL-6 in PTPRO low-expression macrophages through both JAK2 and downstream STAT1 and STAT3. However, these previous studies did not address the mechanism that results in the decreased PTPRO in cancer cells or immune cells. One exception was a study of breast cancer, which mentioned a dramatic decrease in PTPRO during tumor growth due to high methylation in its promoter area.^37^ We found a relationship between increased serum IL-6 levels and decreased PTPRO expression in monocytes and that sustained upregulation of IL-6 increased c-MYC expression and activation through STAT3 activation. We found that the monocyte PTPRO was significantly associated with tumor size and tumor number, mainly due to its related increased PD-L1 expression, and potentially induced immunosuppression.

The increased activation of c-MYC promoted the transcription of miR-25–3 p by binding to its promoter region. As a transcription factor, c-MYC plays roles in cell-cycle progression, apoptosis, and cellular transformation. Constitutive expression of c-MYC has been discovered in carcinomas of the cervix, colon, breast, stomach, and lung.^38 39^ Recently, abnormal expression or activation of c-MYC was reported in immune cells, indicating that c-MYC regulates the TME, thereby activating angiogenesis and suppressing the host immune response.^40–42^ For example, c-MYC inactivation in an immunocompromised RAG1^−/−^ host (deficient for B and T cells) or a CD4^−/−^ host (deficient in CD4^+^ T-helper cells) reduced the kinetics of tumor regression, increased minimal residual disease, and promoted inevitable tumor recurrence.

Activation of c-MYC can suppress the host immune response by causing dysfunction of T and B cells, as well as macrophages.^43^ For example, Pello *et al* reported that c-MYC upregulates the IL-4 signaling mediator STAT6 and that inhibition of the peroxisome proliferator-activated receptor γ, which is also expressed in TAMs, blocks expression of protumor genes, including *VEGF*, *MMP9*, *HIF-1α*, and *TGF-β*.^44^ Our results confirmed a relationship between c-MYC and TAM differentiation and function, and we also revealed that the TAM-promoting effects of c-MYC are miR-25–3 p dependent.

We also found that miR-25–3 p downregulated PTPRO expression in human monocytes, and that transcription of miR-25–3 p was c-MYC dependent. The location of miR-25–3 p is in the miR-106b-25 cluster (encoding miR-106b, miR-93, and miR-25), which is commonly regarded as a proto-oncogenic PTEN-targeting miRNA cluster.^45 46^ Other studies have also indicated that this miRNA cluster could be regulated by c-MYC, in agreement with our results. The miR-106b-25 cluster appears to play an important role in HCC, as it is overexpressed during the development of cirrhosis and subsequently during the development of HCC.^47 48^ We previously reported that miR-25–3 p is an HCC-originated miRNA that can be secreted into the serum and that miR-25–3 p levels were increased >25-fold in patients with HCC when compared with healthy controls.^49^ In the present study, we found increased levels of miR-25–3 p in HCC monocytes, which we postulate to have originated from two sources: from monocytes (promoted by c-MYC activation) and from exosomes. This idea will require verification in future studies.

To date, the characteristics of miR-25–3 p have been rarely addressed, apart from studies that have suggested its potential as a diagnostic biomarker for some malignancies, including papillary thyroid carcinoma, HCC, prostate cancer, and osteosarcoma.^40 45 50–53^ A recent study of liposarcoma revealed that miR-25–3 p could be secreted from tumor cells and that it promoted IL-6 secretion in TAMs via exosomes, but this is the only mechanistic study published concerning the roles of miR-25–3 p in the TME.^44^ Our findings support a role for miR-25–3 p as an inflammation-related miRNA that contributes to immunosuppression by targeting PTPRO transcription in monocytes and TAMs.

## Conclusions

**Figure 7:**
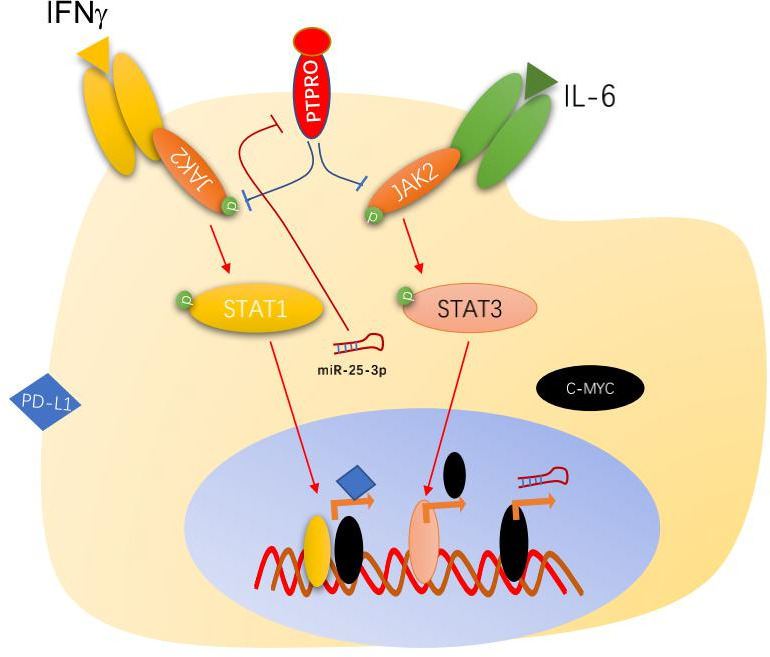
Schematic diagram of protein tyrosine phosphatase, receptor type O (PTPRO)–related PD-L1 upregulation in macrophages. The increased serum IL-6 in patients with hepatocellular carcinoma activated STAT3/c-MYC signaling in peripheral monocytes, enhanced the transcription of miR-25–3 p, suppressed PTPRO expression, promoted PD-L1 through both JAK2/STAT3/c-MYC and JAK2/STAT1 signaling in tumor-infiltrated macrophages, and promoted tumor growth by enhancing T-cell exhaustion.

These results revealed one potential mechanism for the increased PD-L1 expression in monocytes or macrophages within human HCC tumors. The increased serum IL-6 activated STAT3/c-MYC signaling in peripheral monocytes, enhanced the transcription of miR-25–3 p, suppressed PTPRO expression, promoted PD-L1 through both JAK2/STAT3/c-MYC and JAK2/STAT1 signaling, and promoted tumor growth by enhancing T-cell exhaustion (figure 7). We speculated that the PTPRO level in monocyte of patients with HCC was an ideal predictive index in the prediction of the therapeutic effect of anti-PD1/PD-L1 therapy.
