# Supplementary material for: IL-6 promotes PD-L1 expression in monocytes and macrophages by decreasing protein tyrosine phosphatase receptor type O expression in human hepatocellular carcinoma
Source: J Immunother Cancer. 2020 Jun 24;8(1):e000285. doi: 10.1136/jitc-2019-000285 (PMC7319788; doi:10.1136/jitc-2019-000285)
Supplement: Supplementary data [file jitc-2019-000285supp001.pdf]

IL-6 promotes PD-L1 expression in monocytes and macrophages by decreasing protein tyrosine phosphatase receptor type O expression in human hepatocellular carcinoma

Supplementary materials

Supplementary Table 1 The clinicopathological relevance analysis of monocyte PTPRO expression in HCC patients.

	Monocyte PTPRO		
**Variable**	**Low**	**High**	***P* value***
**All cases**	82	83	
**Age**			**0.0081**
<60	32	50	
≥60	50	33	
**Gender**			0.872
Male	52	51	
Female	30	32	
**HBV**			**<0.0001**
Positive	43	68	
Negative	39	15	
**Differentiation grade**			0.358
Well	23	32	
Moderate	32	28	
Poorly	27	23	
**Tumor Size(cm)**			**<0.0001**
≤5cm	20	67	
>5cm	62	16	
**Tumor Number**			**0.0002**
Solitary	57	34	
Multiple	25	49	

* Data was analyzed by chi-squared test. P value in bold indicated statistically significant.

Supplementary Table 2: Primers used in the experiments

Gene	Primer sequence (5'-3')	Amplicon size
Human		
PTPRO	ATGACTTCAGCCGTGTGAGAT	111
	GGGTGGCAATATACTCCTGGG	
CD274 (PD-L1)	TGGCATTTGCTGAACGCATTT	120
	TGCAGCCAGGTCTAATTGTTTT	
Mouse		
Ptpro	GCACACTTTTAATTGGACTGCTC	87
	TGCCAGCTCCACATTCCCTA	
Cd274 (pd-L1)	AGTATGGCAGCAACGTCACG	88
	TCCTTTTCCCAGTACACCACTA	

Supplementary table 3. Information of Antibodies used for flowcytometry analysis

Gene	Manufacturer	Antibody	Clone
**Mouse**			
Pd-L1	BD Bioscience	BD Pharmingen™ APC Rat Anti-Mouse CD274	MIH5
Tim3	BD Bioscience	BD Pharmingen™ PE Mouse Anti-Mouse CD366 (TIM-3)	5D12

Ifn-γ	BD Bioscience	BD Pharmingen™ APC Rat Anti-Mouse IFN-γ	XMG1.2
F4/80	Biolegend	FITC anti-mouse F4/80 Antibody	BM8
**Human**			
CD68	BD Bioscience	BD Pharmingen™ FITC Mouse Anti-Human CD68	Y1/82A
TIM3	BD Bioscience	BD Horizon™ PE-CF594 Mouse Anti-Human TIM-3 (CD366)	7D3
PD-L1	BD Bioscience	BD Horizon™ PE-CF594 Mouse Anti-Human CD274	MIH1
CD14	BD Bioscience	BD Pharmingen™ Alexa Fluor® 488 Mouse Anti-Human CD14	M5E2

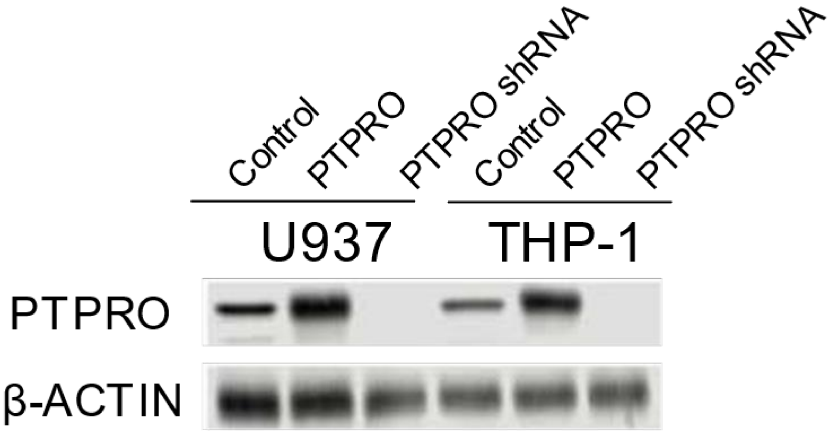

Figure s1: The protein was extracted from cells indicated in the figure and the expression of PTPRO was detected by western-blot.

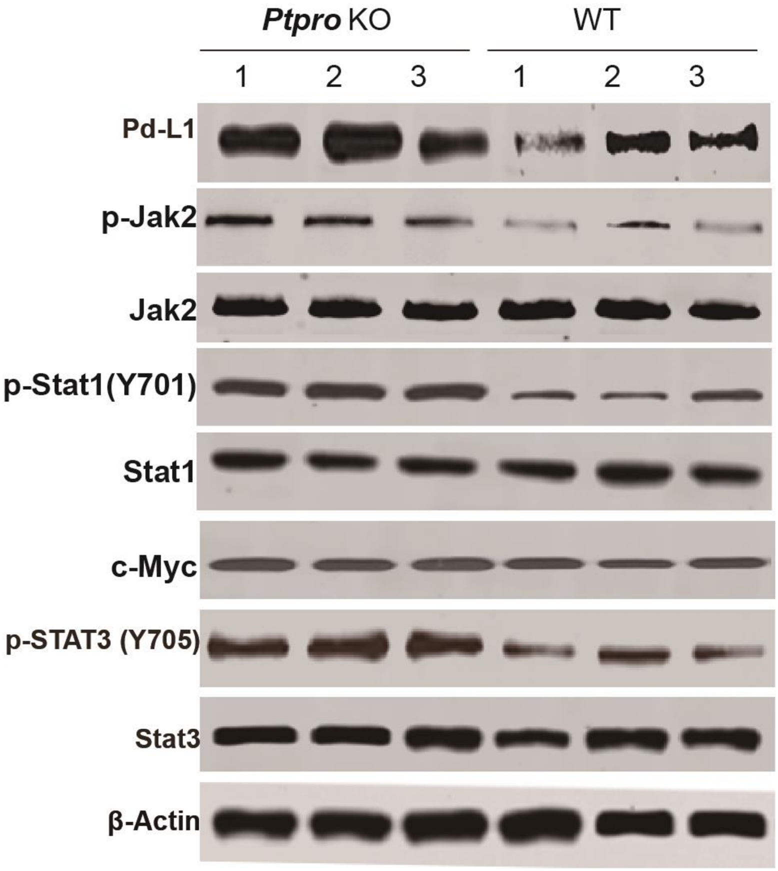

Figure s2: The expression of Pd-L1 as well as Jak2/Stat1 and Jak2/Stat3/c-Myc signaling were detected by western-blot in WT and *Ptpro* KO mice.

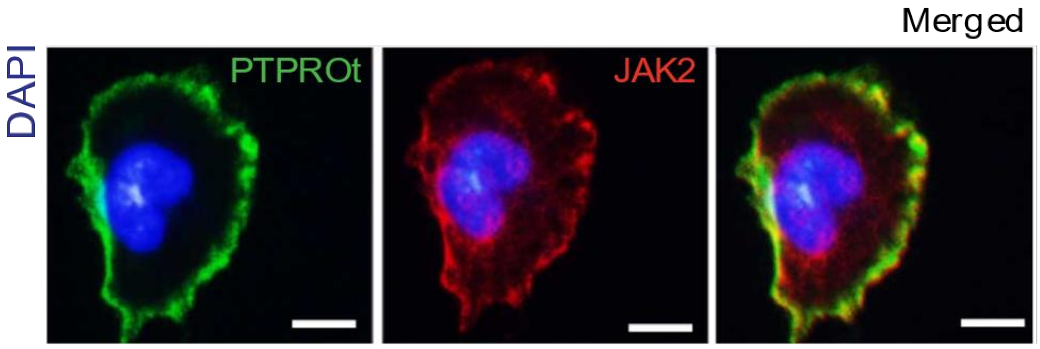

Figure s3: The localization of PTPRO and JAK2 were detected by IF staining (×400).

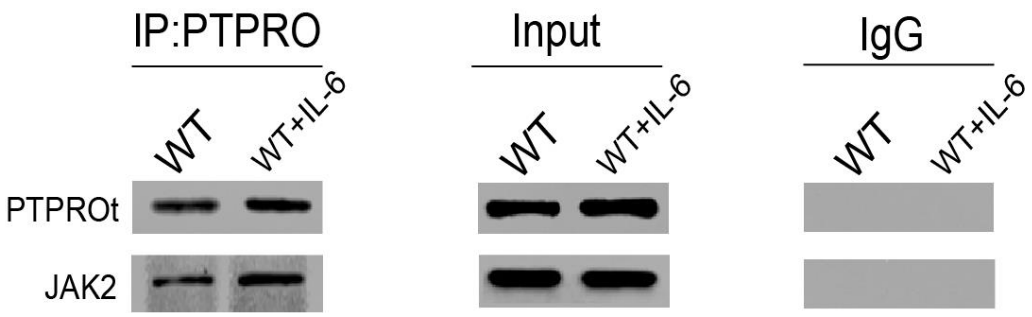

Figure s4: A co-IP assay was performed in macrophage from WT mice treated with or without IL-6 (50ng/mL) by PTPRO antibody, and further detected by using PTPRO and JAK2.

agccatatgggtctgctgctgactttttatatgttgtagagttatatcaagttatgtcaagatgttcagtcaccttga
agaggcttttatcagaaaggggacgcctttctgataaaggttaaggggtaaccttaagctcttacccctctg
aaggtaaaatcaaggtgcgttcagatgttggcttgttgtaaatttctttttttattaataacatactaaatgtggattt
gctttaatcttcgaaactcttcccggtgaaaatctcatttacaagaaaactggactgacatgtttcactttctgtttc
atttctatacacagctttattcctaggacaccaacactagatacctaaactgaaagcttccgccgatttcaccg
aaggtcaggaaagtccaacgcccggcaaactggatttgctgccttgggcagaggtgggcgggaccccgc
ctccgggcctggcgcaacgctgagcagctggcgcgtcccgcgcggccccATGAGGATATTTGCT
GTCTTTATA…

Stat1/STAT3 binding site

Stat2/STAT5 binding site

ATG transcription starting site

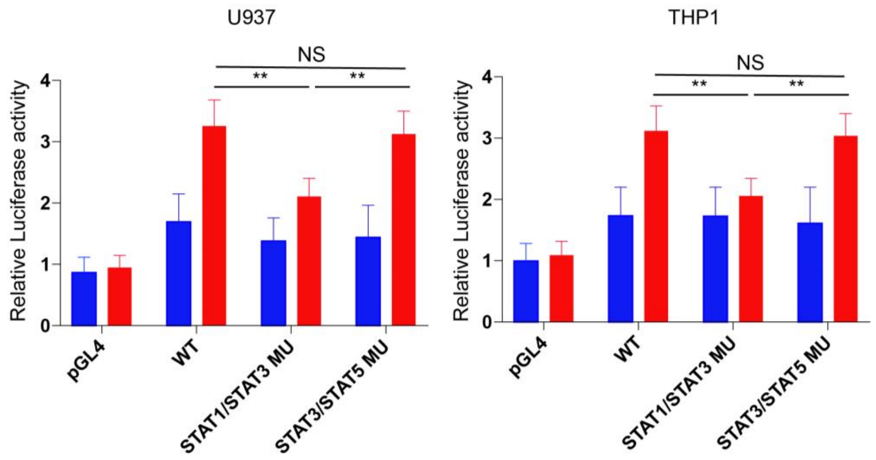

Figure s5. Sequence of human PD-L1 promoter (-500) showing the position of the most representative putative binding sites of STAT1/STAT3, STAT2/STAT5 (upper panel), Luciferase gene reporter assay reflecting promoter activity of PD-L1 treated differently indicated in the figure (Lower panel).

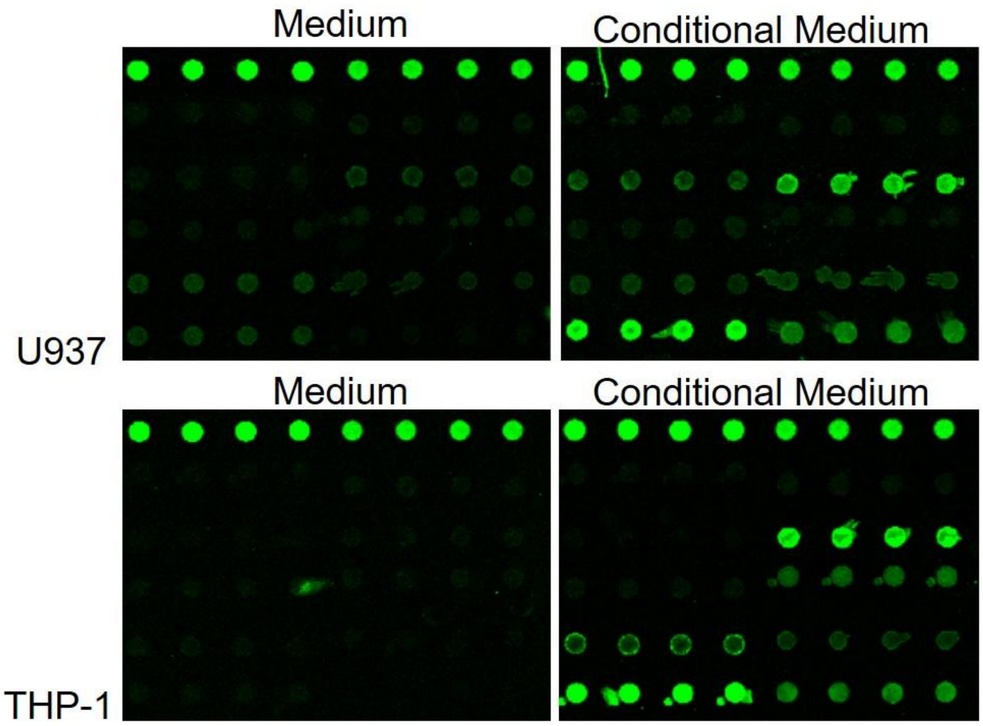

Figure s6: The secretory cytokines detection by human inflammatory array Q1 in the culture supernatant from U937- and THP-1- derived macrophage treated with control and tumor conditional medium.

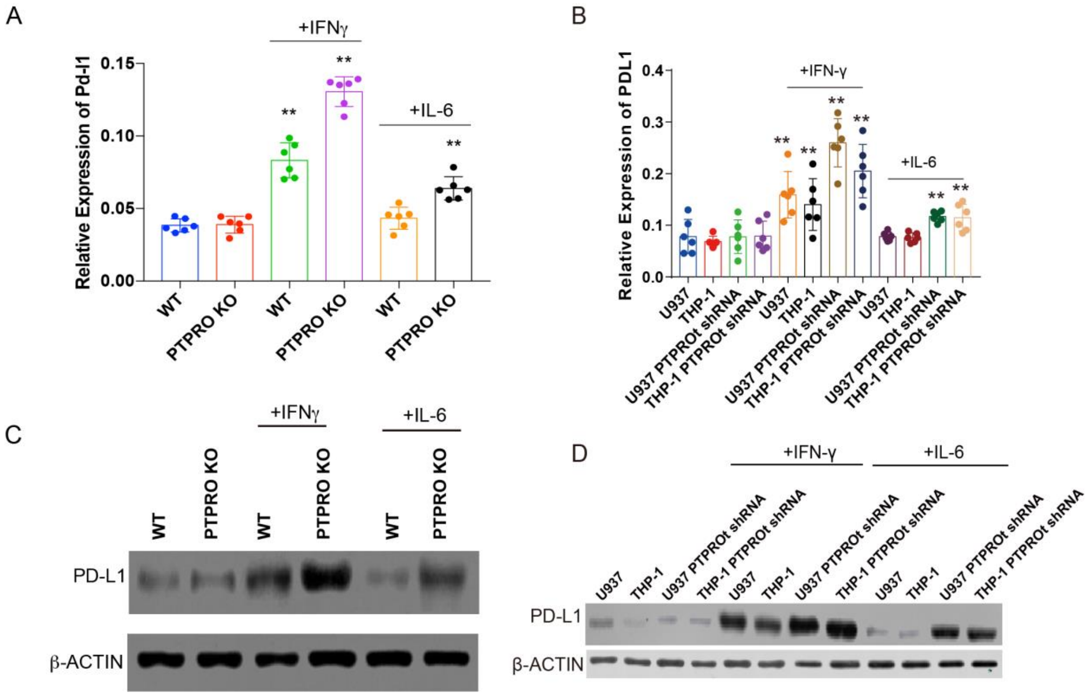

Figure s7: A and B: Macrophages extracted from WT and PTPRO KO mice and human macrophage derived from U937 and THP-1 were treated with IL-6 and IFN- γ , the transcription of Pd-L1/PD-L1 was detected by real-time PCR.**, P<0.01, compared to its no treatment control, by Student's t-test. C and D: Macrophages extracted from WT and PTPRO KO mice and human macrophage derived from U937 and THP-1 were treated with IL-6 and IFN- γ respectively, the protein expression was detected by using western-blot.

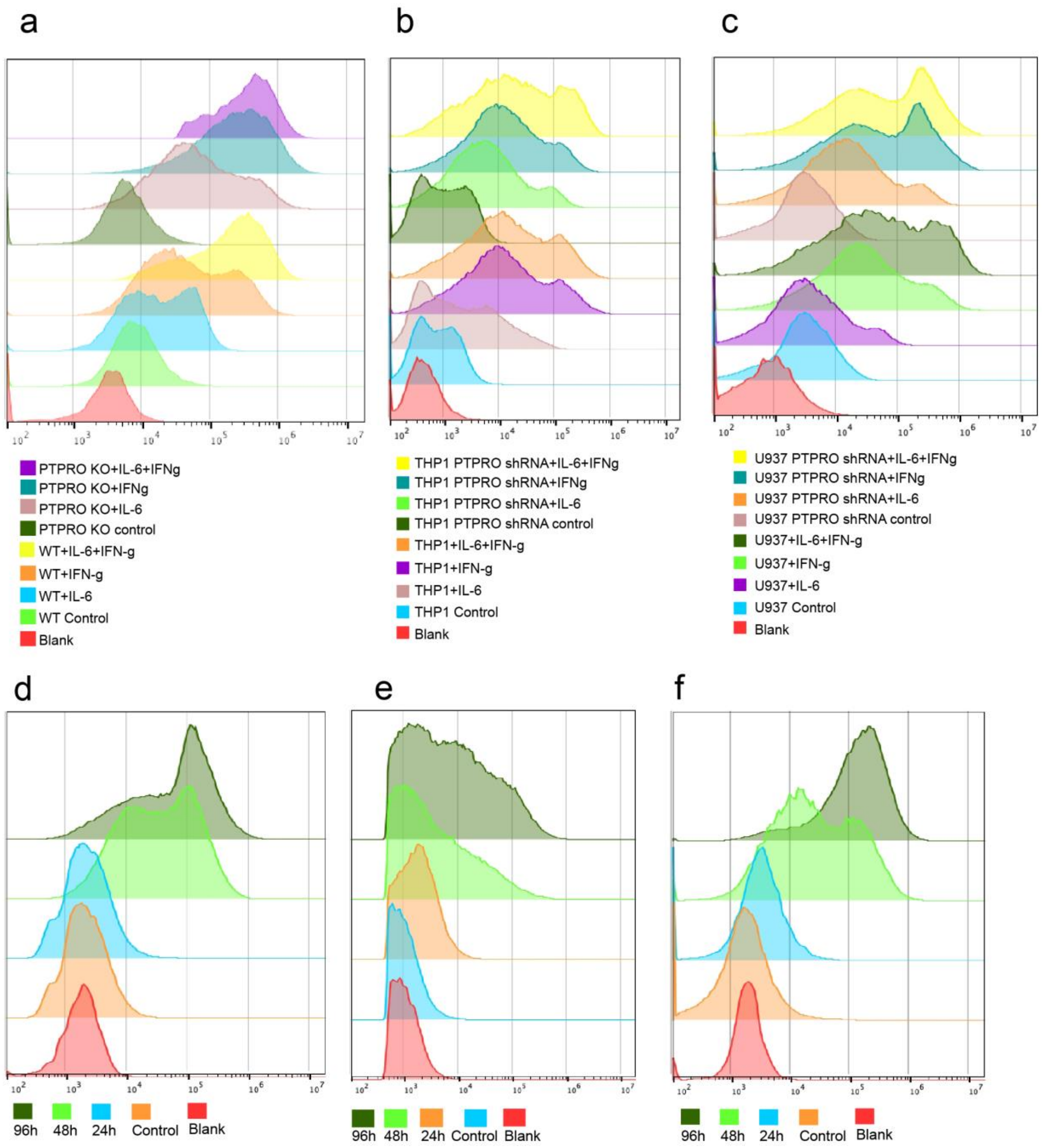

Figure s8. Detection of surface PD-L1 expression in macrophage

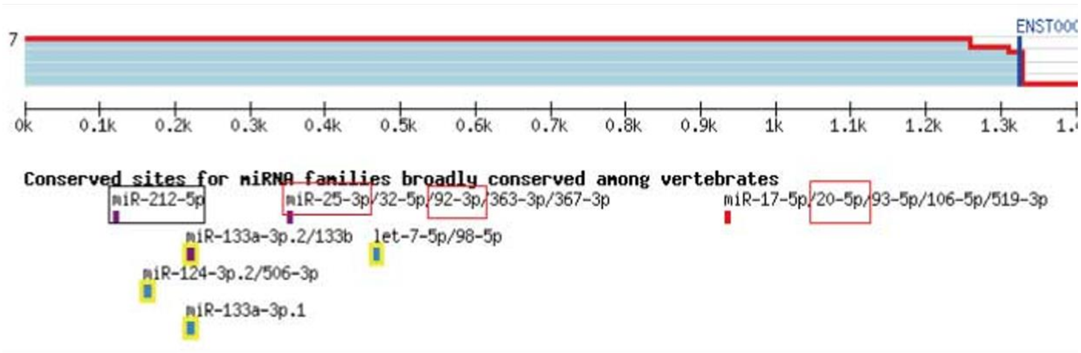

Figure s9: The potential binding sites of miR-25-3p in 3'UTR of PTPRO were predicted by Targetscan.

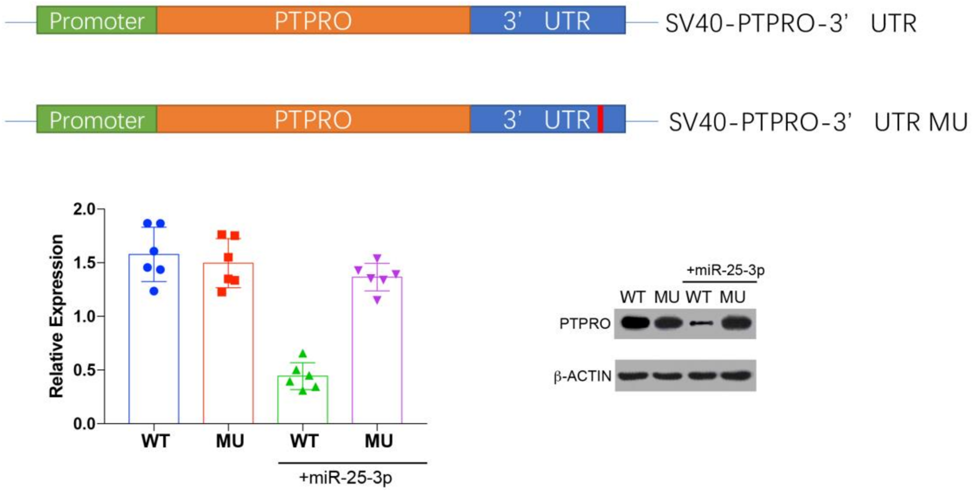

Figure 10. The effect of miR-25-3p on the transcription and protein expression of PTPRO in macrophage.

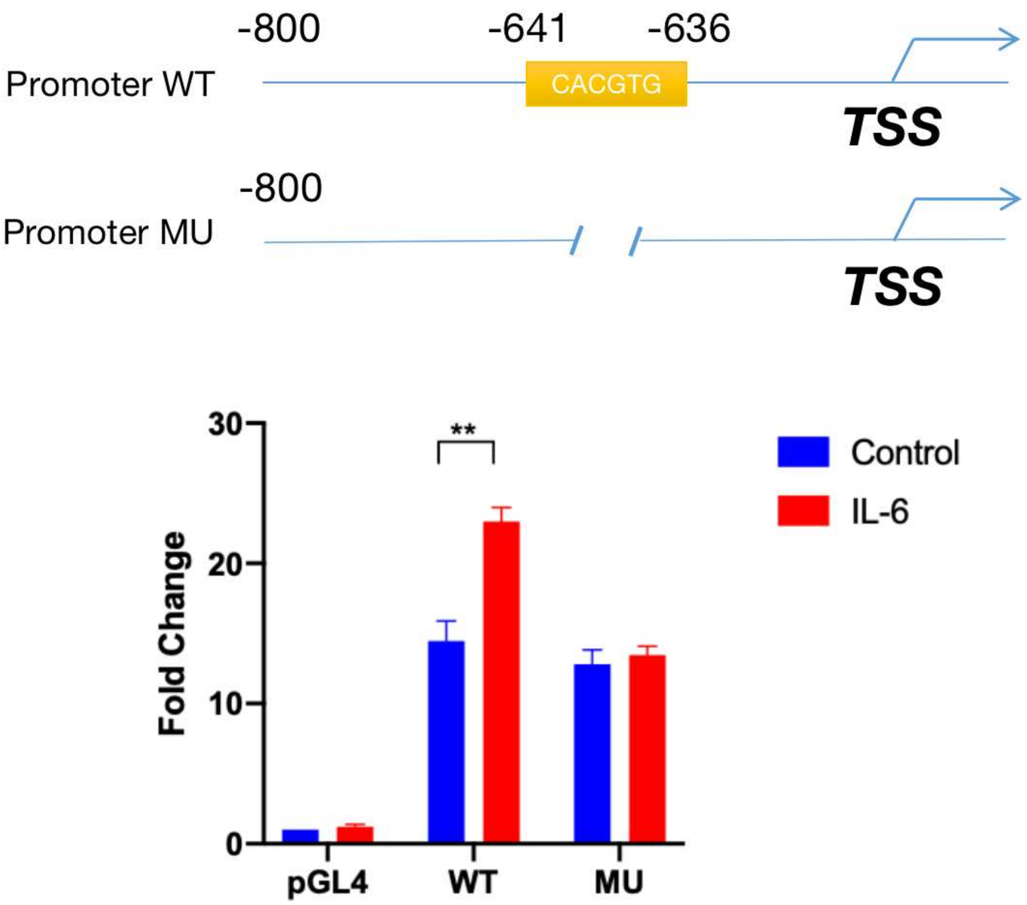

Figure s11 Upper: Schematic representations of genomic regulatory regions of the miR-106b ~25 clusters containing putative c-Myc binding sites. Lower: Luciferase gene reporter assay was carried out reflecting the promoter activity of miR-106b-25 cluster. Error bars represent standard errors from three independent experiments, each conducted in triplicate. **, significantly different ($P < 0.01$).

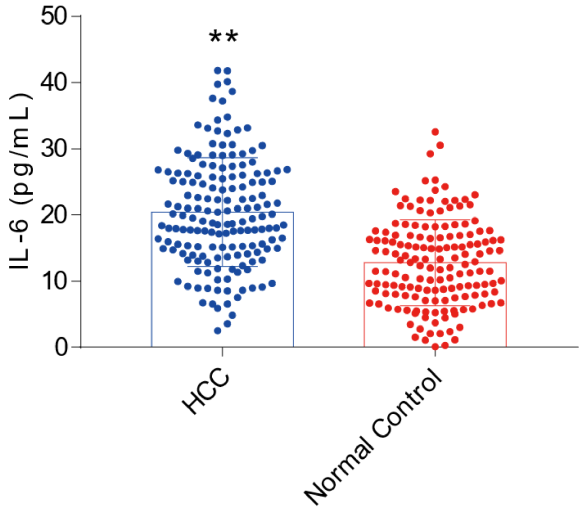

Figure s12: The serum IL-6 of 165 HCC patients and 155 healthy controls were detected by ELISA. **, P<0.01, compared to Normal control, by Student's t-test.

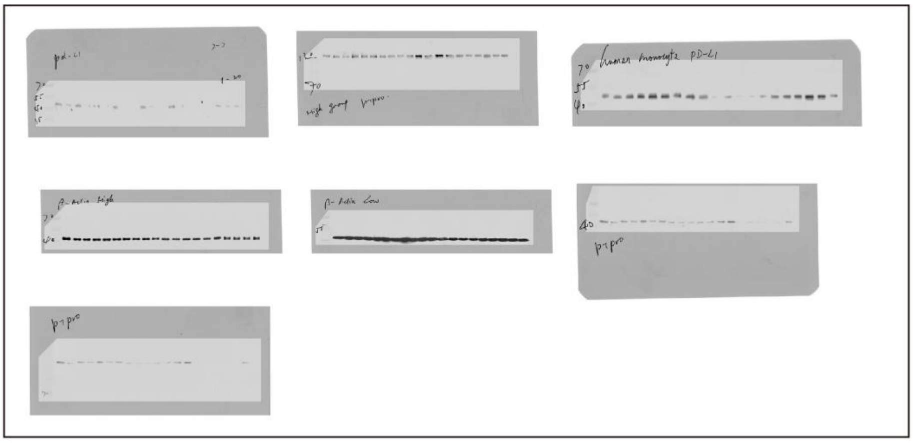

figure 2a

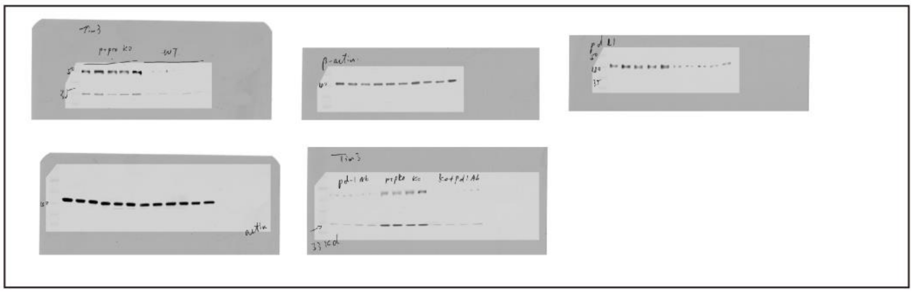

figure 2l

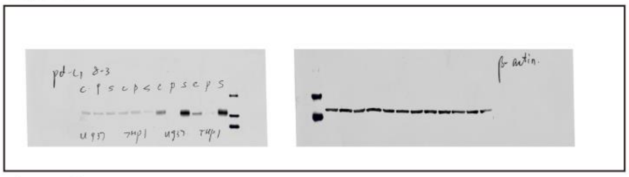

figure 3a

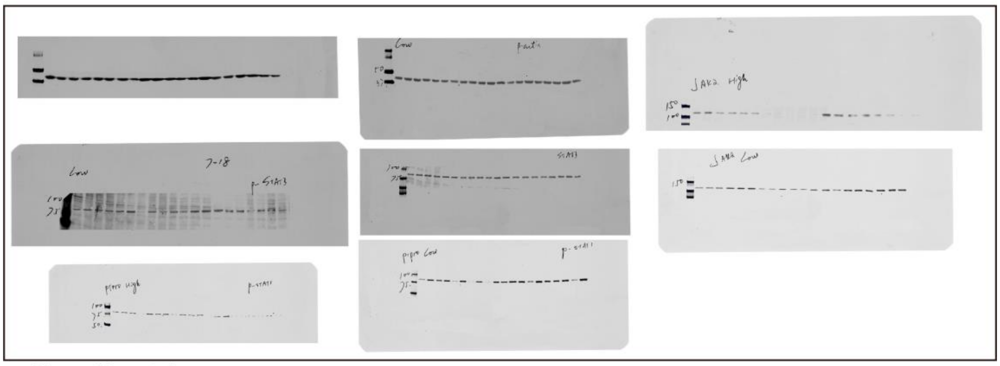

Figure 3b part 1

Uncropped figure of WB

Figure s13

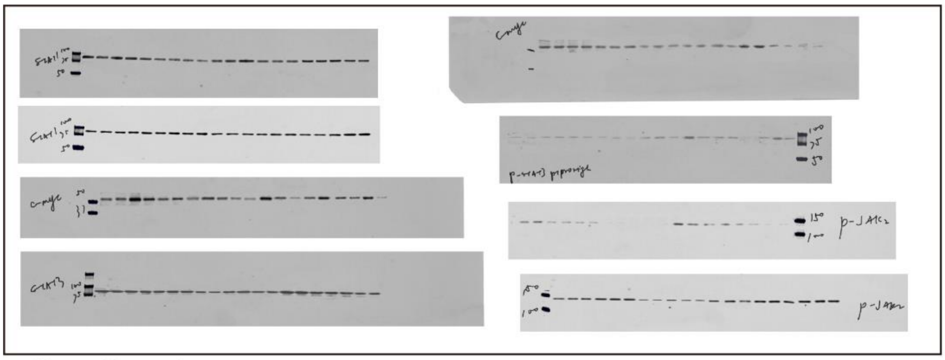

Figure 3b part 2

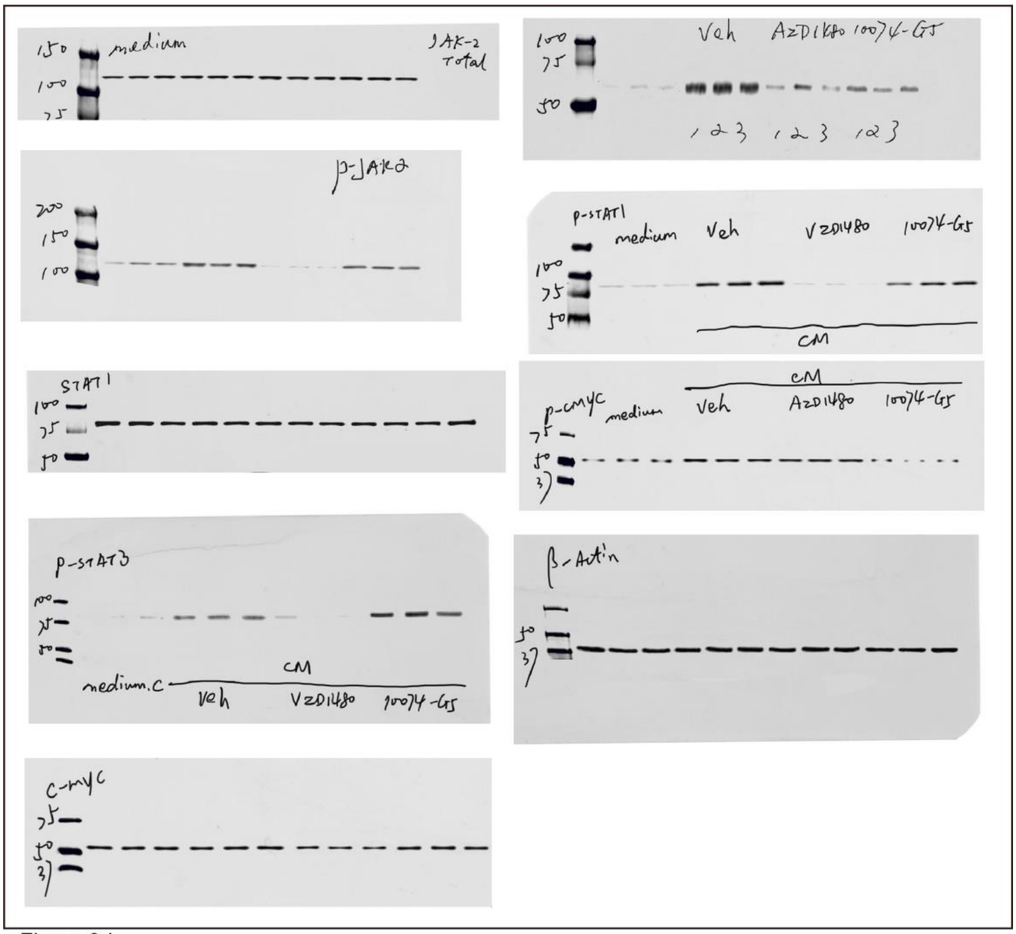

Figure 3d

Uncropped figure of WB

Figure s14

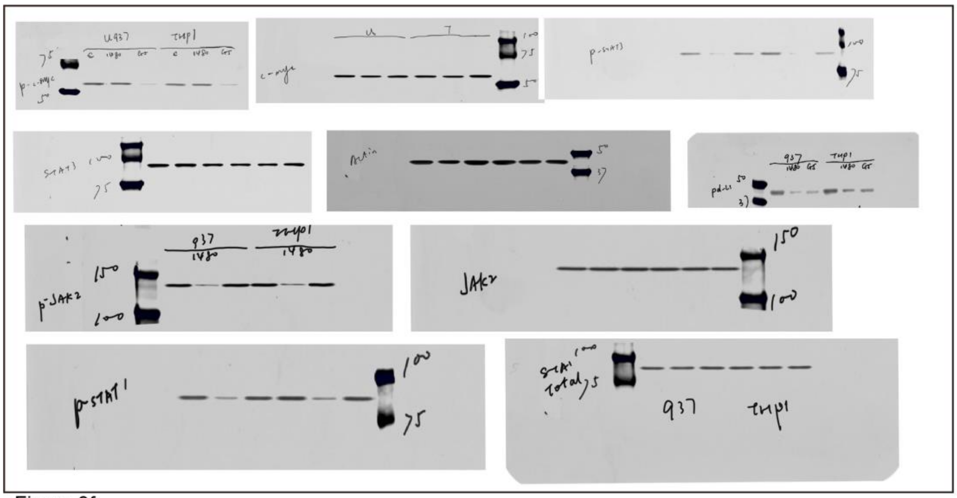

Figure 3f

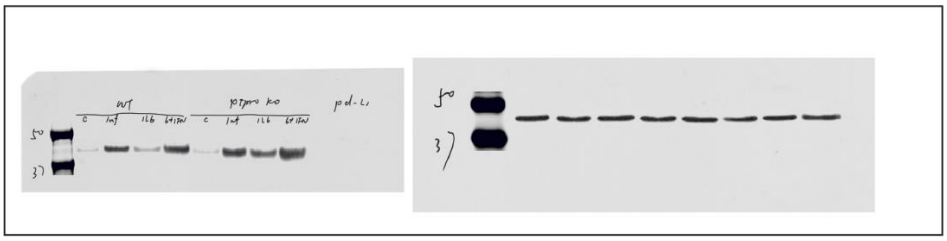

Figure 4a

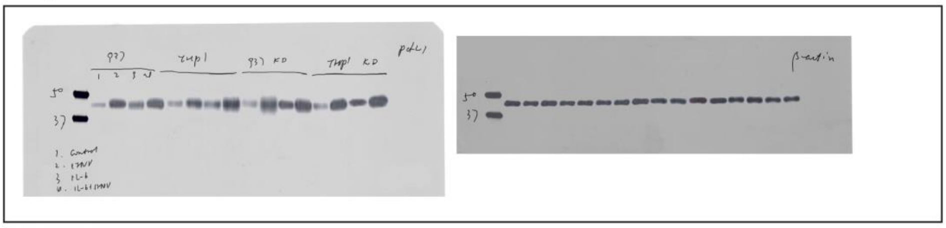

Figure 4b

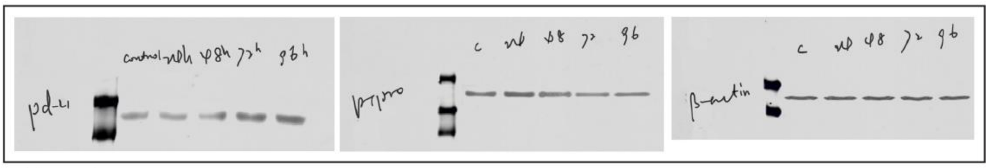

Figure 4c

Uncropped figure of WB

Figure s15

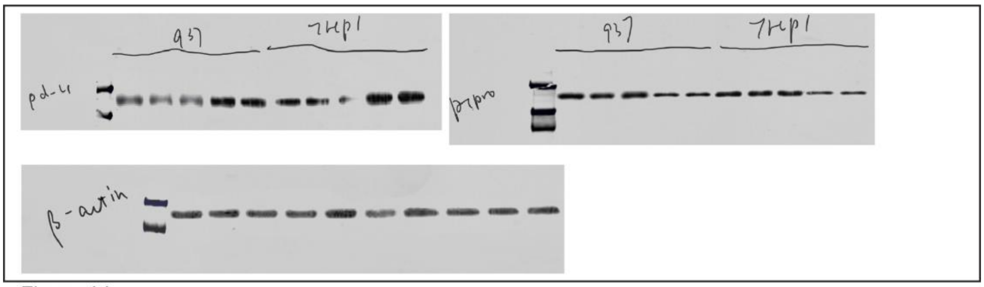

Figure 4d

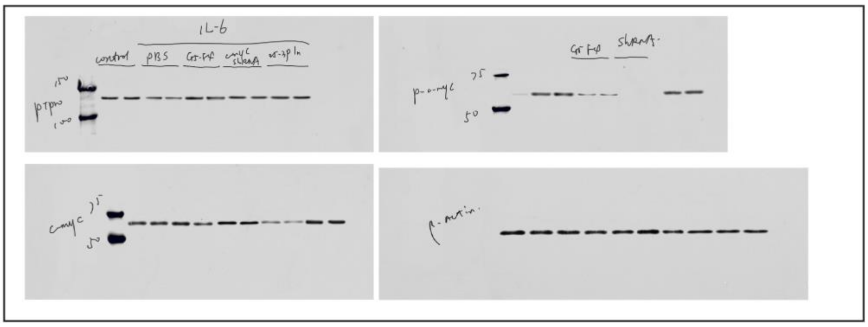

Figure 5h

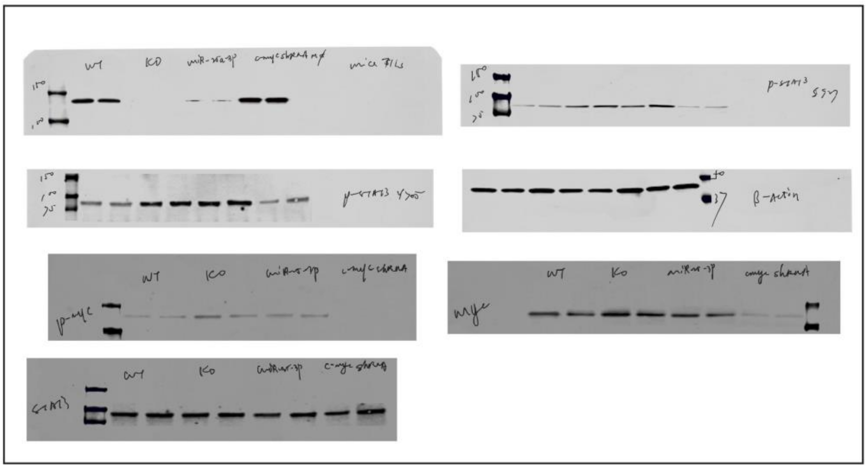

Figure 6d

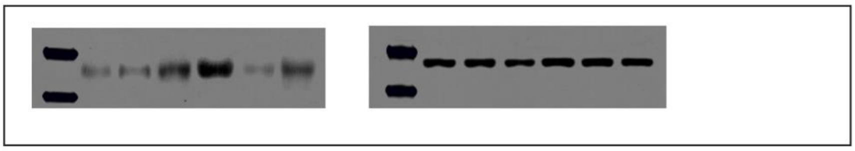

Figure s7 A C

Uncropped figure of WB

Figure s16
